# A systematic review of the barriers to and facilitators of the use of evidence by philanthropists when determining which charities (including health charities or programmes) to fund

**DOI:** 10.1186/s13643-020-01448-w

**Published:** 2020-08-27

**Authors:** Caroline Greenhalgh, Paul Montgomery

**Affiliations:** School of Social Policy, University of Birmingham, Edgbaston, Birmingham, B15 2TT UK

**Keywords:** Barriers and facilitators, Philanthropy, Evidence

## Abstract

**Background:**

Philanthropists, charity leaders and policy-makers have increasingly recognised that the process of giving resources needs to be grounded in evidence—sometimes referred to as ‘evidence-based’ or ‘data-driven’ philanthropy. Yet few philanthropists practise evidence-based philanthropy, and some contend that there is insufficient evidence on which to base their funding decisions. This review aims to identify factors that promote or limit the use of evidence by philanthropists and to rigorously evaluate all existing research on this issue.

**Objectives:**

To identify, synthesise, and evaluate appropriate and rigorous research, examining factors which act as barriers to or facilitators of the use of evidence by philanthropists.

**Methods:**

This review was conducted according to Cochrane standards and reported following PRISMA guidelines. The review protocol was pre-registered (dx.doi.org/10.17504/protocols.io.wbsfane). We searched 10 interdisciplinary databases using a highly sensitive search strategy, developed in consultation with an information scientist. We also contacted experts and searched a range of websites. Studies were included if they comprised primary research into or systematic reviews of the barriers to and facilitators of the use of evidence by philanthropists or funders when determining which charities (including health charities or programmes) to fund. All studies were appraised for quality, and the results synthesised using thematic analysis.

**Results:**

Of 686 studies identified through database and hand searching, nine met inclusion criteria. The thematic summary identified three main barriers to philanthropists or funders using evidence: (1) inadequate knowledge transfer and difficulties accessing evidence, (2) challenges in understanding the evidence and (3) insufficient resources. The three key factors that expedite the use of evidence are (1) improved knowledge transfer and more accessible/relevant high-quality information, (2) access to professional advisors and networks and (3) broadening the definition of what counts as credible evidence along with standardisation of reporting.

**Conclusions:**

The authors of this review found several compelling arguments for promoting the use of evidence by philanthropists to inform their philanthropy. If evidence-based philanthropy is to flourish, then they recommed the following actions: Firstly, philanthropy should be underpinned by a commitment to 'do no harm'. Secondly, the definition of evidence should be expanded and funding decisions based upon consideration of 'all available evidence'. Finally, there should be more investment in synthesizing evidence and in the infrastructure for knowledge transfer.

## Background

It is widely accepted that evidence regarding whether or not an intervention ‘works’ is critical to and should underpin all health and social initiatives and in so doing ensure avoidance of harm. However, despite increasing recognition by philanthropists and funders alike, that the process of allocating funding to charities ought to be underpinned by evidence, few philanthropists practise evidence-based philanthropy in the UK [1]. If donors are to recognise and support the most effective philanthropic programmes, they need to be encouraged to practise evidence-based philanthropy, lest they inadvertently fund programmes that are ineffective or—at worst—actually cause harm. An absence of evidence may also lead donors to fund programmes that already have ample reserves and therefore do not need funding [2]. Our unequivocal support for evidence-based philanthropy is founded in our belief that we should ‘first do no harm’ and a concern that there are multiple ways in which philanthropy can commit unintentional harms. An example of such harms was revealed by The Public Administration and Constitutional Affairs Committee (PACAC) of the House of Commons, which scrutinised the collapse of *Kids Company*, a large charity that had attracted considerable funding from both private philanthropy and the public sector. PACAC raised concerns about safeguarding commenting that ‘There are a number of safeguarding issues which have come to PACAC’s attention during the conduct of this inquiry into *Kids Company*….’ ([3] p. 52). PACAC also concluded that it was not possible to reconcile the claims made by *Kids Co* vis a vis its caseload with evidence from other sources. ‘The evidence is that the figures [relating to outcomes] were significantly over- inflated… [and] was misleading to donors’ ([3] p.51). While we acknowledge that there are many varied and valuable motives which underpin philanthropy, we nevertheless believe that harm reduction is an unequivocal need that is best served by utilising evidence to ensure that the programmes and interventions funded by philanthropy are beneficial to the communities they are intended to serve.

Today, there is growing awareness among philanthropists, charity leaders and policy-makers that the process of giving resources needs to be grounded in high-quality evidence. Accordingly, we have seen the emergence of both ‘evidence-based’ and ‘strategic’ philanthropy, as philanthropists seek to be more outcomes-focused in their giving. Philanthropists and funders are also increasingly recognising that collaboration, sharing knowledge and ‘learning from mistakes’ are a good practice ([4] p.6). The PACAC report: ‘The collapse of Kids Company: lessons for charity trustees, professional firms, the Charity Commission, and Whitehall’ is one such example of ‘learning from mistakes’ as it ‘sought to identify the lessons to be learned from the collapse of Kids Company’ ([3] p.6). Yet it is still rare for philanthropists to ‘draw upon the full extent of available knowledge’ ([5] p. 1).

How donors direct their money to charities matters today more than ever, as government funding to the voluntary sector has declined in both the UK and USA. In the UK, the voluntary sector experienced a fall in government funding of £1.9 billion (down from £15.2 billion to £13.1 billion) between 2009 and

2013 ([4] p.5). Moreover, the National Council for Voluntary Organisations (NCVO) has predicted that there will be an annual shortfall of ‘£4.6 billion …. in sector income over the next five years, simply to maintain current spending power’ ([6] p.6) by 2019. In the USA, non-profits have experienced similar funding shortfalls as they have had to contend with a decline in funding from both state and federal governments in tandem with changes to the tax code, both of which have squeezed corporate giving [7]. If charities are to retain their independence and ensure a sustainable funding base, they will need to seek funding from alternative sources, which in many cases will be from philanthropists. Certainly, ‘… philanthropy has been on the rise since the financial crisis, with 2016 seeing the highest amount given’ in the last decade ([8] p.15). In light of the increasingly competitive funding environment, such a rise in donations by philanthropists is both significant and of particular interest to charities. Accordingly, the way in which donors practise philanthropy and how they use evidence in their decision making needs to be explored. Findings of such research can be disseminated to charities to better enable them to develop and manage their relationships with such donors and to access their financial support.

While the need to generate evidence to support philanthropic funding decisions is clear, the extent to which philanthropists will use it is less certain. The utility of evidence may depend on how readily available it is to those making funding decisions, whether or not philanthropists can distinguish between the different qualities of evidence, and whether the available evidence is relevant to their question and aligns with their own tastes and preferences.

Moreover, what is meant by ‘the best available evidence’ is contested, particularly in light of the differing types and weight of evidence in the social sciences [9, 10]. Greenhalgh [11] refers to a ‘hierarchy of evidence’ (shown as a pyramid) which ranks randomised control trials (RCTs) and systematic reviews at the top of the pyramid and situates ‘expert’ opinion and qualitative research at the bottom. In reality, the type of evidence that will prove the most useful in determining the best way to address a particular problem will, to an extent, be determined by the nature of the question being asked [9]. Quantitative research, for example, may be best placed to answer questions relating to the extent to which something works (such as ‘how many?’ or ‘how much?’) whereas qualitative research may be better placed to answer how and why something works, as the purpose of qualitative research is to ‘explore people’s perceptions and experiences of the world around them’ ([12] p.2).

### Rationale

There is limited research on how donors use evidence to inform their philanthropy and on the barriers to and facilitators of their use of evidence. To date, much of the research in this area has concerned the extent of giving by donors rather than how they choose charities. Studies that do examine the manner in which donors choose charities usually focus on the ‘why’―namely, the donor’s motivation for choosing the charities―rather than the ‘how’, with its focus on the mechanisms by which donors choose charities.

This systematic review seeks to address this gap in the research by identifying the factors that may limit or promote the use of evidence by philanthropists and by evaluating existing research on this issue. This will help support the development of mechanisms to address the barriers and scale up those factors that facilitate evidence-informed philanthropy. Our rationale is that enhanced access to and understanding of high-quality evidence in tandem with improved communications and sharing of knowledge will enable philanthropists to make better judgements which in turn will lead to ‘better and more sustainable philanthropy’ ([4] p.16). This will give philanthropists confidence that they are funding effective initiatives and interventions that will ultimately lead to the change they seek, for ‘if one role of philanthropy is to solve the tough problems … we need all the answers we can get’ ([13] p.2).

### Objectives of the review

To identify, evaluate and synthesise appropriate and rigorous research, examining factors which act as barriers to or facilitators of the use of evidence by philanthropists.

## Methods

This review of the barriers and facilitators to the use of evidence by philanthropists and funders was conducted according to the standards of Cochrane [14] and Campbell reviews [15]. The protocol for this review was assessed by two specialists in issues related to the third sector, to ensure that the methods and search strategy were exhaustive. The final search string was developed in consultation with an information expert (File 4). The protocol was published at (dx.doi.org/10.17504/protocols.io.wbsfane).

We did a highly sensitive search involving electronic resources, hand searching and contacting experts and initially found nineteen studies. Each of those studies was critically appraised using GRADE CER-qual, and nine studies were included in the final data synthesis. Many of the barriers and facilitators were unique, although others were reflections of each other.

**Fig. 1:**
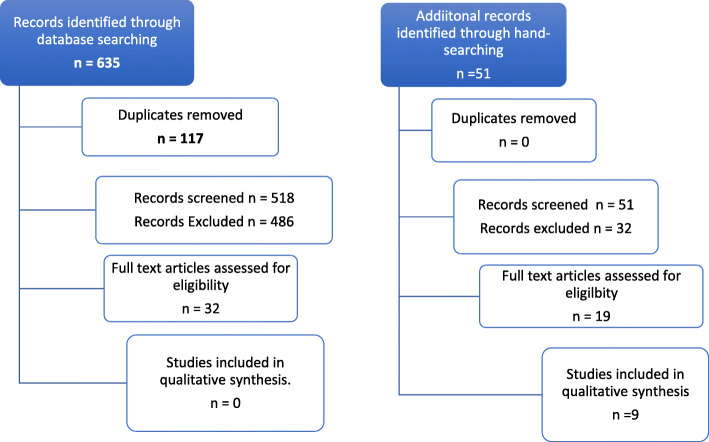
PRISMA Flowchart capturing flow of studies through the review

This systematic review utilised both PRISMA guidelines, which stipulate a minimum standard for describing the findings of systematic reviews (see Fig. 1 PRISMA Flowchart capturing flow of studies through the review), and also employed the GRADE-CERqual (Grading of Recommendations Assessment, Development, and Evaluation - Confidence in the Evidence from Reviews of Qualitative research) approach—see Tables S4 to 9. The GRADE-CERQual tools permit us to ascertain the level of confidence that we can have in our findings by employing four components comprising methodological relevance, coherence, adequacy and relevance to determine the level of confidence that we can have in the findings arising from individual reviews within syntheses of qualitative evidence. Finally, we utilised the JBI Critical Appraisal Checklist for Qualitative Research [16] to appraise each of the included studies (see Additional File 5).

### Search strategy

In conducting a systematic review of the qualitative literature, inadequate cataloguing of qualitative research means that despite combining terms and employing precise and wide-ranging search strategies pertinent studies may still be overlooked ([17] p. 5). As such, we used a highly sensitive search strategy to capture all extant research on the barriers and facilitators experienced by philanthropists seeking to utilise evidence. The following databases were searched:

1. ABI/INFORM Global
2. Applied Social Sciences Index and Abstracts (ASSIA)
3. International Bibliography of the Social Sciences (IBSS)
4. PAIS Index
5. Policy File Index
6. Social Services Abstracts
7. Social Science Premium Collection
8. Worldwide Political Science Abstracts
9. SCOPUS
10. Open Grey
11. ProQuest Dissertation and Theses Global

The authors anticipated that due to a lack of rigorous, independent research to provide an authoritative basis for our understanding of philanthropic activity [18], it would be a challenge to uncover much literature relating to the use of evidence by philanthropists. To address these limitations and also to

minimise publication bias, the authors utilised supplementary search methods which they blended with database searching. Supplemental search methods employed included hand searching of journals and the bibliographies of relevant articles, contacting experts and searching relevant websites including:

- Philanthropy Impact
- New Philanthropy Capital
- The Lilly Family School of Philanthropy
- Ten Years’ Time
- UBS Philanthropy
- Charities Aid Foundation (CAF)
- Nonprofit Quarterly
- Candid (comprising Foundation Centre and GuideStar)
- Association for Fundraising Professionals
- Institute of Fundraising

### Study selection

To be eligible for inclusion, studies must have been published in English and be primary research or systematic reviews investigating the perceptions and/or experiences of philanthropists, high-net-worth individuals (HNWIs) or funders (including grant-making organisations) relating to their use of evidence. All study designs were eligible for inclusion provided they examined factors affecting the use of evidence by philanthropists; however, such factors need not have been the primary focus of those studies. Studies investigating the perceptions of professionals (such as charity CEOs, philanthropic advisors and philanthropic consultants) as to how philanthropists (or how they perceive philanthropists to) use evidence were also eligible for inclusion. Articles were initially screened at the title, publication date and abstract level by CG using Endnote.

### Population

The primary populations of interest were philanthropists, high-net-worth individuals and funders (including grant-making organisations) who make significant donations (sometimes referred to as major gifts) to fund charities or third-sector organisations (which include voluntary organisations, community-based organisations and non-profits). The secondary populations of interest were third-sector practitioners (such as charity CEOs), philanthropic advisors and philanthropic consultants.

### Definitions

This review adopted the following definition of ‘philanthropist’: an individual who makes donations to charities and non-profits with the intent of addressing social problems [19]. ‘Funders’ comprise grant-making organisations, such as trusts and foundations, which award financial grants to charities and non-profits.

This review defined ‘barriers’ as things or circumstances that impede the ability of philanthropists (or charity professionals) to use evidence to inform their philanthropy or grant-making. ‘Facilitators’ are defined as any factors or conditions that enhance the ability of philanthropists (or charity professionals) to use evidence to inform their philanthropy or grant-making. Barriers and facilitators do not need to be the primary outcome of interest of a study to be considered for inclusion.

### Screening and data extraction

Studies were stored, screened and coded using NVIVO software, and all data were extracted by CG with a 20% random sample screened independently by PM. Any disagreements were resolved by consensus.

Extracted data from the final set of included studies were captured in a data extraction table (Table S2); a further table (Table S3) captured the characteristics of the excluded studies (see Additional file 1). The tables were developed by the two authors to record the following information:

- Year of publication
- Author
- Title
- Country where study was conducted
- Study aims

## Methods

- Population (philanthropists, foundations or charity practitioners)

### Key findings:

- Identified barriers to utilisation of evidence
- Identified factors of utilisation of evidence

Tables S4 and S5 present the methodological limitations of the included studies as they relate to each finding, using GRADE CER-Qual methods. Table S6 captures the data from individual studies that contributed to each review finding. Table S7 is a CER-Qual Quality of Evidence Profile, while Table S8 summarises our qualitative findings. Finally, Table S9 summarises our confidence in each of the individual studies. Tables S4 to S9 are attached in Additional file 2.

### Data synthesis

The main purpose of this review was to identify limiting and promoting factors regarding the use of evidence as identified by philanthropists, funders and charity practitioners. Consequently, the review includes studies comprising a variety of research designs, namely quantitative, mixed methods and qualitative. In light of this, we conducted a thematic analysis to integrate the data, through which the main findings and theories were extrapolated and then ordered as either barriers or facilitators to form a narrative synthesis [20, 21]. We then listed factors identified as being either barriers or facilitators (regarding the use of evidence by philanthropists) and analysed their frequency. Ideally, simple ‘vote-counting’ techniques should be avoided in research as they increase the risk of bias by not typically considering study methods or study quality [22]. We have mitigated this risk by employing thematic analysis to order the individual factors and identify variations in factors ‘revolving around the same underlying problem’ ([23] p.2).

### Quality assessment

Prior to data synthesis, the authors employed a number of critical appraisal tools to assess the credibility and rigour of the included studies and to ensure transparency in the appraisal process. However, many critical appraisal tools require the reviewers to score each of the studies against specific criteria, so studies that do not report all of the criteria will score low even though they might not merit it [24]. Hence, quality appraisal (particularly across different study designs) necessitates an element of judgement on the part of the researchers.

While this was first and foremost a narrative exercise, for studies that employed a qualitative research design, we conducted methodological assessment using the Joanna Briggs Institute’s Critical Appraisal Checklist for Qualitative Research [16]. The single quantitative study in our review was assessed utilising a critical appraisal checklist adapted from Crombie [25], and the two mixed-methods studies were appraised using the Mixed Methods Appraisal Tool (MMAT) version 2018 (http://mixedmethodsappraisaltoolspublic.pwbworks.com/w/file/fetch/127916259/MMAT_2018_criteria-manual_2018-0801_ENG.pdf).

The included studies were subjected to the GRADE CER-qual assessment [12] to ascertain the level of confidence that we could have in our findings. The methodological limitations of each of the included studies are reported in Table S8 (see Additional file 2); the methods of data collection and analysis and any limitations that arose in relation to each of the specified themes are reported in Tables S4 and S5 (see Additional file 2). In addition, we highlight the contributions made by the individual studies to each of the research findings and themes, providing insight into whether particular studies contributed more or less to the thematic framework in Tables S6 and S7 (see Additional file 2).

The level of confidence awarded to each study was informed by the methods of data collection and analysis that were used, the level of methodological rigour and the total number of items fulfilled on the appraisal checklists. Studies were then ranked as either high, medium or low quality. We then used our own informed judgement, taking into consideration the value of the insights derived from the individual study alongside the study’s methodology, as those “rated as ‘low quality’ because of methodological flaws or lack of reporting may nevertheless generate new insights, grounded in the data…” ([24] p.375).

## Results

The 51 records that emerged from the supplemental hand searching were combined with 635 records which were identified through the database searching; together, these amounted to 686 records. After removing 117 duplicates, 518 studies from the database were screened at title and all 51 studies from the supplemental search were screened at title (569 in total). Four hundred eighty-six studies from the database search and 32 studies from the supplemental search were excluded following scrutiny of the title and in some cases abstracts. Thirty-two studies identified through database searching were retrieved as full text, and 19 studies identified through the supplemental search were retrieved in full. Of these, a further 42 were excluded for the following reasons: not being primary research or systematic reviews focusing on the experiences of philanthropists, funding organisations, third-sector professionals, philanthropist advisors and/or consultants; or for not being primary research or systematic reviews concentrating on the barriers to and facilitators of the use of evidence. Nine studies were included in the final synthesis. None of the included studies emerged from the database searches; they were all derived from the supplemental search.

### Characteristics of included studies

The characteristics of included and excluded studies are presented in Tables S2 and S3 (see Additional file 1). Of the nine included studies, one was purely quantitative, two used mixed methods and six were purely qualitative. The majority (five) were conducted in the UK (55%); two were conducted in the USA (22%); and one study (11%) comprised a series of nine workshops conducted in seven countries: Dubai, Ecuador, India, Malaysia, Singapore, the UK and the USA. Eight of the studies were published between 2010 and 2018 (88%), and one was published in 2003 (11%).

### Populations of the included studies

Three of the included studies had a sample size of more than 100: one administered a questionnaire to 3254 people; another sampled more than 200 participants, each of whom participated in a series of workshops; and the third study sampled more than 500 participants. Six studies had a sample size of less than 100, ranging from fewer than 10 to 99. Participants in the studies were philanthropists, including high-net-worth individuals (two studies); philanthropists and philanthropic advisors (one study); philanthropy advisors and consultants together with charity practitioners (one study); funders comprising trusts and foundations (two studies); philanthropy practitioners and consultants (one study); academics, funders and professionals working in the non-profit sector (one study); and academics, charities, NGOs, advisors, businesses and professionals (one study).

### Quality of the included studies

We appraised each of the nine included studies using GRADE CER-qual methods [12] and further evaluated the six qualitative studies using the *JBI Critical Appraisal Checklist for Qualitative Research* [16]. The single quantitative study was appraised using a version of the Crombie tool [25], and the two mixed-methods studies were appraised using the MMAT version 2018 (http://mixedmethodsappraisaltoolspublic.pwbworks.com/w/file/fetch/127916259/MMAT_2018_criteria-manual_2018-0801_ENG.pdf). Two studies were deemed to be of high quality, four of medium quality and three were assessed to be of low quality.

### Identified barriers and facilitators

All nine of the studies described at least one barrier to the use of evidence, although this was not confined to the use of evidence by philanthropists. Eight of the studies described at least one facilitator of the use of evidence. Twenty-seven unique factors were identified as barriers to the use of evidence and thirty-three factors as facilitators of the use of evidence. In a number of instances, the barriers and facilitators were reciprocal, for example:

- Too much information and insufficient synthesis of information were recognised as barriers by two of the studies [study no. 4 and 7], and improved and more readily available synthesis of evidence was identified as a facilitator by one of the studies [study no. 9].
- Insufficient knowledge dissemination and lack of availability of and access to evidence was deemed to be a barrier by five studies [study no. 2, 4–7], and five studies [study no. 2, 3, 5, 7, 9] identified knowledge dissemination, knowledge hubs and open data as facilitators of the use of evidence.
- One study [study no. 5] reported that a paucity of measurement tools proved to be a barrier to the use of evidence, and the same study reported that the provision of appropriate measurement tools would serve to facilitate the use of evidence.

The top three barriers to the use of evidence identified by philanthropists and funders included difficulties in accessing relevant and high-quality evidence (six studies). This was illustrated by one study which pointed out that the main types of evidence relied upon by funders (such as data synthesis and scoping reviews) ‘are different to the main types of evidence they generate (e.g. impact reporting) and share with others (e.g. evaluations)’ ([26] p.5). Problems relating to philanthropists’ and funders’ understanding of the evidence (three studies) and insufficient resources to identify and use the evidence (six studies) served as further challenges to engaging with evidence.

The factors cited most frequently as facilitating the use of evidence were better knowledge transfer and improved ease of access to evidence (six studies). Recognised mechanisms for knowledge transfer included ‘peer-to-peer sharing of experience and practice’ ([27] p.10); philanthropists and funders could also engage more ‘proactively with the new What Works centres, both to share evidence for dissemination, and to seek evidence that will inform their funding’ ([26] p.5). The provision of open data and feedback loops also aided knowledge sharing and improved ease of access to evidence. Access to professional advisors and experts (three studies) and a broader definition of what counts as credible evidence along with better standardisation of reporting (three studies) also facilitated the use of evidence.

### Thematic analysis

The nine included studies comprised a range of research designs, including qualitative, quantitative and mixed methods. By conducting a thematic analysis, we took an inductive, data-driven approach, which helps with both the extraction and interpretation of the complex data. Moreover, ‘the use of thematic analysis ensures credibility as it is transparent, rational and uniform, allowing the reader to have confidence in the findings’ ([28] p.22). This approach is in line with common practice when aggregating data from different types of research. As there is no ‘gold standard’ in conducting systematic reviews on barriers and facilitators, we drew on published examples of this type of review including Bach-Mortensen and Montgomery [23] and Oliver et al. [20]. Specifically, all identified factors were identified and organised into barriers and facilitators and counted by frequency. The identified factors were then categorised following thematic analysis, thus enabling the synthesis to account for the arbitrary difference of factors revolving around the same underlying problem.

Our review comprised nine studies describing at least one barrier or one facilitator and included one survey, two mixed-methods studies and six qualitative studies. Table 1 illustrates which studies contributed to the identified barriers and facilitators.

**Table 1:** Barriers and facilitators and participant numbers

	Quantitative study *N* = 3254 Fidelity study [4]	Mixed methods studies *N* = 923 Carrington study [2]—40 participants Tillotson study [8]—500 participants + 383 professional services firms	Qualitative studies *N* = 306 Breeze study [1]—60 participants Kail Study [6]—9 participants Ravenscroft Study [7]—13 participants Van Poortvliet Study [9]—12 participants David & Lucille Packard Study [3]—12 participants Jones et al. [5]—200 participants
Difficulty accessing evidence Studies 1, 2, 3, 4, 5. 6, 7, 9	80% (*n* = 2604) expressed concerns that there was not sufficient relevant information to determine the credibility and trustworthiness of a non-profit or what the impact of their donation is.	*n* = 40	*n* = 306
Challenges in understanding the evidence Studies 1, 2, 5, 7, 9	65% (*n* = 2115) did not know the impact of their funding.	N/A	*n* = 285
Insufficient resources Studies 1, 2, 5, 7. 8, 9	N/A	*n* = 923	*n* = 285
Knowledge transfer and ease of access Studies 2, 3, 5, 6, 7, 9	65% of (*n* = 2115) would give more if they had at least one insight into the impact of their giving.	n = 40	*n* = 246
Professional advisors Studies 2, 6, 8	N/A	*n* = 923	*n* = 9
Broader definition of what counts as credible evidence and standardisation of reporting Studies 2, 5, 7, 9	N/A	*n* = 40	*n* = 225

### Synthesis of findings

All nine of the included studies described at least one barrier to the use of evidence, although this was not confined to the use of evidence by philanthropists. Eight of the included studies described at least one facilitator of the use of evidence.

A number of the barriers and facilitators reported in the studies were labelled differently despite their having similar underlying constructs, for example, lack of skills and insufficient staff may be part of the same underlying problem as a lack of resources. To deal with this, we organised all of the factors into six overarching categories, comprising three barriers and three facilitators:

1. Difficulties in accessing evidence (six studies)
2. Challenges in understanding the evidence (three studies)
3. Insufficient resources (six studies)
4. Knowledge sharing and ease of access (six studies)
5. Professional advisors and networks (three studies)
6. A broader definition of what counts as credible evidence and better standardisation of reporting (three studies).

Table S6 extrapolates the data from each study supporting each category (see Additional file 2).

The most commonly cited barrier to using evidence was difficulty accessing it. The reciprocal theme that was reported most frequently as a facilitator comprised those factors relating to sharing knowledge and ease of access.

## Discussion

This systematic review examined the barriers to and the facilitators of the use of evidence by philanthropists and funders when deciding what to fund. It found that structural considerations including ease of access to high-quality evidence alongside relevance and ease of comprehension were key determinants of whether or not evidence was used.

The presumption that philanthropists seek to make pragmatic, evidence-informed decisions may not always be the case as ‘philanthropic donors are often misled by nature and by their instincts’ [2]. It may be that many donors do choose to apply evidence, but they do so only in a limited way, for although most donors are motivated by a desire to make a difference, many will already have aligned themselves to a particular cause before reading the evidence or conducting or commissioning research. By electing to support a charity that matters to them personally, rather than using evidence to identify which causes are most effective, donors may overlook more effective charities and interventions and inadvertently support organisations that have less impact (https://www.effectivealtruism.org/articles/introduction-to-effective-altruism/). Hence, what motivates philanthropists to give to one particular genre of charity over another might determine the extent to which they will seek to use evidence to underpin their giving.

Two key themes emerged as barriers to the use of evidence: difficulty in accessing relevant and high-quality data and a lack of understanding of that information. Further analysis revealed that the evidence sought and relied upon by philanthropists was rarely aligned with the evidence generated by the beneficiary charities. Moreover, much of the data that are generated by charities is deemed to be largely irrelevant to donors [26]. This is somewhat ironic given the often ‘reported tendency’ of many charities and non-profits to ‘customise their evaluation’ and reporting procedures to satisfy their funders ([23] p.10).

Another constraint relating to the use of evidence is the cost of obtaining relevant evidence. Few philanthropists and funders are willing to fund the cost of evaluations of interventions for example. Consequently, a number of studies, including our own, have highlighted the absence of impact measurement among TSOs. Despite the relatively transparent nature of British charity regulation, only a small minority of charities report on their impact to the Charity Commission [29] and a significant proportion state that they do not measure impact at all [30]. Most TSOs need help in collecting and analysing data which they can use to minimise harm and maximise effectiveness. A review of 24 previous studies conducted by one of the authors, of the barriers and facilitators to impact measurement [23] found that the most common barriers to engagement in evaluation were lack of expertise and internal capacity, mismatch between funder requirements and what TSOs perceived to be appropriate evaluation goals and the lack of financial resources to conduct evaluation. The factors most often reported as facilitators included involving stakeholders in identifying relevant outcome indicators and evaluation goals, having the appropriate training of staff to engage in evaluation and having the motivation to understand and improve the effectiveness of the delivered services. In reviewing 55 studies of the adoption of Evidence Based Interventions (EBIs) by TSOs, Bach-Mortensen et al. [31] found that the most frequently reported barriers were related to recruitment and retention of service-users, problems in adapting EBIs, lack of financial and human resources, and implementation difficulty. Facilitating factors included issues related to organisational culture (e.g. whether the EBI matched the mission of the TSO), flexibility and resources for TSOs to implement the EBI, perceived effectiveness of the EBI, organisational support and prioritisation and supportive leadership.

Further barriers relating to accessibility of evidence included a lack of scholarly research on the area in question; a lack of access to such research (particularly if it is stored behind a paywall, in which case the cost might be a deterrent); poor organisation of research; and on occasion the sheer volume of information. These problems are compounded by insufficient dissemination of high-quality and synthesised knowledge. Resource constraints emerged as an additional barrier to the use of evidence, including a lack of people, time and funding. Indeed, the cost of accessing scholarly research along with the requisite time required to ‘search for and identify relevant research’ may mean that such research is only used when it is deemed essential ([32] p.5). A lack of skills and tools to appraise the quality and reliability of evidence (including critical appraisal skills) and relevance (wrong or insufficient information) were also cited as barriers to using evidence.

The consequences of not using evidence were revealed in the only quantitative study in our review. It revealed that 80% (*n* = 2604) of donors felt apprehensive about the impact of their donation (35 p.4), which manifested as ‘unease about determining an organization’s credibility or trustworthiness’ and ‘frustrations that some non-profits do not always explain how a charitable donation will be used’ ([33] p.9). Moreover, 65% of the respondents agreed that ‘at least one insight into the impact of their giving … would influence them to give more,’ which would imply either that they do not have enough evidence to determine the impact of their donation (on the outcomes of the charity), or they do not have sufficient understanding of the evidence or the skills to appraise it.

Six studies identified inadequate transfer of knowledge and difficulties accessing evidence as barriers. Inadequate infrastructure may be a contributing factor to poor transfer of knowledge, particularly the lack of any single identifiable mechanism for supporting dissemination [34]. Consequently, ‘formidable difficulties’ stand ‘in the way of disseminating the knowledge that is available’ ([5] p.1). These difficulties are exacerbated by a lack of incentives for philanthropists to share, commission or seek out knowledge [35]. Furthermore, some charities may not wish to share knowledge that communicates a failure to achieve their desired outcomes [4] or they may be reluctant to forego a competitive advantage by sharing knowledge [35]. Two studies advised that good practice in sharing knowledge expedites the use of evidence—formal and informal networks can enhance the dissemination of knowledge through collaboration, for ‘many knowledge entrepreneurs in philanthropy get and give their most useful knowledge through peer-to-peer networks’ so ensuring ‘that knowledge has a connection to practitioner problems and needs’ ([35] p.10). Philanthropists and funders can also enable learning and innovation simply by sharing their own data even if they do not have the skills or resources to analyse that data themselves ([26] p.20). Some models of knowledge dissemination are purported to be more effective than others, for dissemination strategies usually concentrate ‘on the supply side of knowledge sharing, rather than the demand side’ and rarely ask the question ‘what knowledge do users need?’ ([35] p.4). Ravenscroft suggests implementing feedback loops from beneficiaries to donors to help further inform the relevance and quality of desired evidence [26] and further recommends engaging proactively with the ‘What Works Centres’ (WWC) to enhance the use of evidence, which could then inform funding decisions [26]. The first of the WWCs was set up by the UK government in 2010 to facilitate access to high-quality evidence regarding what works across a number of fields. The WWCs are intended to aid ‘more effective and efficient services across the public sector at national and local levels’ [36]. So far, WWCs have been established in education, crime, early intervention, local economic growth, ageing and well-being. Similarly, in the USA, the What Works Clearing House (WWCH) provides a central and trusted source of scientific evidence on education interventions. The WWCH uses a systematic review process to identify all of the research on an intervention, assess the quality of each study and summarise the findings from the high-quality studies.

A number of the included studies expressed concerns that a narrow definition of what counts as credible evidence can act as an additional barrier to using evidence; however, several funders specifically raised ‘concerns about the quality of evidence’ ([26] p.16). As highlighted in the background to this review, what is meant by ‘the best available evidence’ is contested, but as Cairney points out, the most useful evidence for determining the best way to address a particular problem will largely be determined by the nature of the question being asked [9]. The David and Lucille Packard Foundation agree, cautioning that ‘information is context specific’; hence, it is important to specify the question ([35] p.7). However, Schorr and Farrow point out that frameworks which tightly determine what is ‘acceptable evidence’ can discourage the use of evidence by limiting available knowledge ([5] p.1). They conclude that there should not be an insistence on absolute proof—their findings reveal that ‘the value of many kinds of interventions can be …. Understood and acted upon without having to be proven through experimental methods’ ([5] p.v). Oliver et al. agree, recommending in their systematic review examining the barriers to and facilitators of the use of evidence by policymakers that ‘all research should be based on an understanding that a broader interpretation of “evidence” than “research-based” evidence is also essential’ ([20] p.9).

Difficulties accessing high-quality and relevant information also hinders the use of evidence. Breeze [37] notes that the sheer volume of data and information available to donors is a challenge, as they lack the resources needed to rationally assess it all. On the other hand, some philanthropists found it hard to access good quality data because it is not readily available [38].

Five of the studies recognised that access to high-quality information or evidence facilitates evidence-informed philanthropy. Indeed, access to high-quality information upon which to base decisions is a pre-requisite to more and better-quality giving, [33] with the quantitative study reporting that 65% of respondents (*n* = 2115) would donate more if they had a better understanding of the impact of their donation. One study identified syntheses of knowledge about ‘what has worked and how’ as a mechanism that facilitates the effective use of evidence and thus serves to ‘make interventions more effective and implementation stronger’ ([5] p.iv). Nevertheless, even when data are available, one study found that philanthropists and philanthropy professionals do not ‘pay as much attention as they could (some might

argue should) to acquiring knowledge that could help them’ ([27] p.5). This is despite the argument that, in view of the fact that philanthropy is subsidised by the Treasury (with the intention of generating public benefit through private giving), philanthropists have a moral obligation or duty to ensure that any decisions they make concerning the distribution of philanthropic funds are ‘based on a full consideration of available evidence’ ([27] p.6) and [39].

The studies in this review identified a number of factors which support the use of evidence, including signposting and the publication of links to research findings [13] through knowledge hubs. The creation of feedback loops [35] can help to assess the appetite for knowledge that is relevant and accessible and to inform researchers of the gaps in the research base. Several studies identified a need to reappraise what is deemed to be ‘credible’ evidence and in so doing enable philanthropists and funders to ‘make use of all the knowledge we can muster—– from multiple sources’ ([5] p.iii), for the ‘idea that nothing is worth knowing unless you know it for certain has its place, but not when applied to complex social programs’ ([5] p.v). One study posited that standardised reporting would facilitate the use of evidence, although that same study acknowledged that ‘measuring the effectiveness of everything from protecting the environment to tackling world hunger on the same terms is tricky. New methodologies such as the Global Impact Investing Rating System (GIIRS) are emerging, but none is yet viewed as a panacea’ ([40] p.35). Irrespective of sector, different Third Sector Organisations currently present outcome measures as they see fit. This is problematic as it limits comparability between providers and prevents the sharing of data collection tools and analysis methods. It may also lead to bias in the choice of outcomes and may crowd out issues that really matter to many stakeholders. Thus, a common set of outcomes (perhaps within sectors, e.g. for elderly care, for child welfare) would allow for comparability and simplicity, and economies of scale for third sector providers. Core outcome sets overcome the measurement problems above, and methods to develop them have been developed by the COMET Initiative [41] to accomplish this goal. While it is accepted that this is not an easy task, it has been achieved in many spheres and has many advantages for the sector and should not be dismissed.

### Limitations

None of the included studies were derived from the systematic search of the data bases, rather they each emerged from the supplemental searching. Consequently, none of the included studies was subject to peer review although it should be recognised that many non-peer-reviewed papers are of high quality and vice versa [42]. Similarly, “scholars are increasingly recognizing instances where it seems appropriate to broaden the evidence search beyond the limits of academic journals to incorporate ‘grey literature’” [43]. Moreover, the included studies were subjected to both PRISMA guidelines and the GRADE-CERqual approach to ensure that we could have confidence in the quality of the included studies.

A further limitation was that the heterogeneity of study designs made it challenging to compare quality across the studies in this review. In addition, some studies used vote-counting techniques to analyse their findings, which involved counting the number of times each factor was mentioned, without any weighting of importance. This makes it difficult to determine the impact of each factor.

The included studies had a range of methodological weaknesses. However, as this was intended to be a narrative review, even the one third of studies graded low quality were analysed, as we deemed them likely to offer valuable insights [24].

We were surprised to find that even those studies that sought to make recommendations relating to the better use of evidence in philanthropy did not employ rigorous methodology (or at least did not convey the methodology they used in their studies: we had to write to the publishers or authors of the individual reports to ascertain their methodology).

### Recommendations for future research

In their study, exploring the ‘Benefits of open access to scholarly research for voluntary and charitable sector organisations’, Beddoes et al. [32] found that a lack of skills and tools to appraise the quality and reliability of evidence (including critical appraisal skills) and relevance (wrong or insufficient information) were barriers to using evidence. This review did not find many references to the skills or tools that would enhance the use of evidence by philanthropists and funders, but it is surmised that certain skills and tools would better equip them to engage with and critique evidence. Future research, therefore, could focus on the use or otherwise of those specific skills and tools and the extent to which they improve both uptake and understanding of evidence.

Two thirds of the studies cited challenges in accessing evidence and research, together with insufficient knowledge transfer, as barriers. Future research should therefore explore ways to enhance the transfer of knowledge and to better understand which research philanthropists and funders deem to be relevant and accessible.

Four of the included studies called for broadening what constitute credible evidence; thus, research that seeks to clarify our understanding of different types and values of evidence would be welcomed.

Two studies revealed the growth in the philanthropy advice market which ‘since the turn of the century’ has emerged ‘with the aim of helping philanthropists give their money away well’ ([2] p.6)*.*

This finding suggests that professional advisors could be a crucial conduit to wealthy donors, and thus, the quality of the advice they offer is significant. As such, some form of professional standards and accreditation for philanthropy advisors would be welcomed in the UK to ensure that the quality of advice is of a minimum standard and is reliable. Indeed, one of the included studies [44] reports that the 12% of philanthropists who take professional philanthropy advice are responsible for 53% of the donations given by high- and ultra-high net-worth individuals in the UK and therefore concludes that philanthropy advisors should be made more readily available. However, this figure only suggests correlation, not causation, as those philanthropists who sought out professional advice may already have been donating a much higher percentage of funds to charities than their peers. Therefore, further research is needed to ascertain the extent to which philanthropic advisors are responsible for increased giving vis-á-vis their clients and also to examine whether those receiving such advice give more effectively than their peers.

## Conclusions

This review has highlighted several compelling arguments for supporting and encouraging philanthropists and funders to use evidence in their decision making. If evidence-based philanthropy is to flourish, then the following steps are recommended.

First, it is imperative that all philanthropy is underpinned by a commitment to ‘do no harm’ (similar to the Hippocratic oath). In January 2019, the National Council for Voluntary Organisations published a statement of ‘Charitable Ethical Principles’ which, while stopping short of a commitment to ‘do no harm’, did state that all ‘charities should proactively champion ethical behaviour and reflect and apply their charitable values in any activity they undertake’ ([6] p.1). However, it is also important that the manner in which we collect and employ evidence (to ensure that we avoid harm) is carefully scrutinised. As recently noted in a report published by the International Committee of the Red Cross, new technologies present both risks and opportunities for humanitarian action and if we are to ‘ensure that their use does not result in any harm, humanitarian organisations must develop and implement appropriate data protection standards, including robust risk assessments’ ([45] p.4).

Second, the definition of evidence should be expanded, and funding decisions should be ‘based on a full consideration of available evidence’ ([27] p.9). Moreover, where there is insufficient evidence, funders and philanthropists should be encouraged to invest in generating new evidence [27]. We should also employ more standardised indicators and measures that can be more widely understood and easier to compare.

If high-quality, widely accepted, readily understood, user-friendly and reliable measures and indicators are to be available where they are most needed, philanthropy … must become more intentional about investment in developing appropriate data sources, indicators, and measures. ([5] p.6)

Indeed, in a recent letter to the Secretary of State for Digital, Culture, Media and Sport, civil society groups (comprising: the Institute for Government, Full Fact, Nesta, the Open Data Institute, mySociety, the Open Knowledge Foundation, the Royal Statistical Society, the Open Contracting Partnership, 360Giving, Open Ownership, and the Policy Institute at King’s College London) warned that a failure to invest in better data means the government is unable to properly understand its own operations and the quality of public services. They urged the UK Government to ‘transform its use of data’, or the UK will fall ‘behind other countries’, and pointed out that ‘the upcoming *National Data Strategy* offers the chance to seize the new data environment, and use it to deliver better public services, and improve the economy and society for future generations’ [46].

A number of foundations are already providing financial support to help set up new specialist research and teaching centres and funding journals and other methods of communicating about philanthropy to a wider audience. Such journals should be open access rather than their content being placed behind a paywall. Attention is also needed to ensure that philanthropists improve their understanding of reputable sources of knowledge or evidence and how to appraise evidence.

Two of the key barriers to evidence-based philanthropy are paradoxical. On the one hand, donors are sometimes confronted by overwhelming amounts of evidence and data that they cannot use effectively due to a lack of time, resources or skills. This calls for more investment in synthesising evidence. On the other hand, a number of philanthropists and funders reported a lack of data or, more specifically, a lack of high-quality evidence relating to the areas they wished to fund. Thus, in addition to encouraging the collection of more knowledge, there is a need for more sharing of existing knowledge through networks and journals, and ‘state- and community-level initiatives’ should be encouraged to ensure that all new programmes ‘generate rigorous new evidence’ in tandem with the ‘development of the tools and capacities’ to ‘help local communities generate new knowledge at greater scale’ ([5] p.6). The rise in What Works Centres both in the UK and similar organisations in the USA illustrate the uptick in acceptance that sound evidence is important to drive policy.

Finally, poor knowledge transfer and the lack of an infrastructure to facilitate such knowledge transfer, particularly when combined with a lack of incentives that might encourage charities, funders or philanthropists to share, commission or seek out knowledge, presents a considerable barrier to the use of evidence. Indeed, many philanthropists have expressed surprise that “compared with some other ‘business’ sectors with which they are familiar, there is less specialist research or knowledge transfer within the philanthropy sector” ([27] p.8). However, the emergence of a new generation of philanthropists (many of whom are giving away money they have made in their lifetime rather than inherited) has resulted in new models of philanthropy informed by their commercial expertise [47], as they theorise that practices employed by the commercial sector are equally appropriate for and transferable to charities [26]. Many such philanthropists not only seek to ensure that their gift is outcomes-focused but also that it is cost-effective and will produce a discernible social return [8]. Hence, they employ mechanisms to measure and evaluate the impact of their donation and as such some philanthropists are already well placed to fill knowledge gaps [27].

In conclusion, we theorise that the practice and impact of philanthropy is considerably enhanced by the application of the learning and knowledge that emerges from evidence-based research. Moreover, when resources are finite and not sufficient to meet the extensive demands made upon them, those responsible for deciding how funding will be allocated should endeavour to ensure that those decisions are informed by detailed consideration of all the accessible evidence [27].

## Supplementary information

**Additional file 1.** Tables of Extracted Data.

**Additional file 2.** GRADE CERQual Tables.

**Additional file 3.** PRISMA checklist.

**Additional file 4.** Search Strategy.

**Additional file 5.** JBI Checklists.

## Data Availability

All data generated or analysed during this study is included in this published article and the supplementary information files.
