# Supplementary material for: A systematic review of the barriers to and facilitators of the use of evidence by philanthropists when determining which charities (including health charities or programmes) to fund
Source: Syst Rev. 2020 Aug 27;9:199. doi: 10.1186/s13643-020-01448-w (PMC7453541; doi:10.1186/s13643-020-01448-w)
Supplement: Supplementary file 1 — Additional file 1. Tables of Extracted Data. [file 13643_2020_1448_MOESM1_ESM.docx]

## **Included Studies**

1. Breeze, B. 2013 *How donors choose charities: the role of personal taste and experiences in giving decisions*
2. Carrington, D. 2009 *The Application of Learning and Research to the Practice of Philanthropy*
3. David & Lucille Packard Foundation 2003 *Marketing your knowledge: A report to philanthropy’s R&D organisations*
4. Fidelity 2017 *Overcoming Barriers to Giving*
5. Jones, T, Dewling, C. Alexander, J. 2018 *Future of Philanthropy Insights from Multiple Expert Discussions Around the World*
6. Kail, A. Johnson, S. & Bowcock, M. 2016 *Giving more and better – How can the philanthropy sector improve?*
7. Ravenscroft, C. 2013 *The Secrets of Success? How charitable funders use and share evidence in practice.*
8. Tillotston, C. 2016 *Learning to give: lessons for advisers and would-be philanthropists*
9. Van Poortvliet, et al.2011 *Foundations for Knowledge: Sharing knowledge to increase impact: a guide for charitable funders*

## **Table 2. Characteristics of included studies**

| **Study No.** | **Author, Year Published, Country** | **Name** | **Description** | **Study Design or Focus** | **Participants** | **Key Findings** |
| --- | --- | --- | --- | --- | --- | --- |
| 1. | Breeze, B.  2013  UK | How donors choose charities: the role of personal taste and experiences in giving decisions | Study seeks to explore the role of personal taste and experience in determining which charities donors choose to support. | Qualitative study utilising semi-structured interviews. | 60 participants - philanthropists | - Decision to donate to a particular charity is primarily driven by the donor’s tastes and personal background. - Inertia and path dependency account for many decisions to support particular charities. - Donors frequently support charities that promote their own preferences despite subscribing to the belief that charitable giving should be directed to the needy. |
| 2. | Carrington, D.  2009  UK | The Application of Learning and Research to the Practice of Philanthropy | Study explores the availability and use within Europe of research into philanthropy and social investment and how a stronger and more effective framework could be built to enhance and extend opportunities for study and for learning in order to improve the practice of philanthropy. | Mixed methods study combining qualitative methods utilising semi-structured interviews and a quantitative audit. | 40 participants drawn from academia and research | - Perception that the philanthropy sector is uninterested in and unwilling to pay for research into their own behaviour, effectiveness or impact; and for practitioners to perceive academics as being prone to the ‘over collection’ of data and inclined to be dismissive of many of the questions that practitioners would like studied. - Neither the philanthropy sector nor the academics that study it were sufficiently committed throughout their practice to knowledge transfer |
| 3. | David & Lucille Packard Foundation 2003  USA | Marketing your knowledge: A report to philanthropy’s R&D organisations | Explores how good ideas are circulated in philanthropy. | Qualitative study in which 12 participants were interviewed. | Interviewed 12 individuals working in philanthropy and worked with 10 participant organisations. | A number of obstacles—in norms, structure and practices are a barrier to accessing evidence. Some of these obstacles are systemic. Others are caused by poor practice and can be changed. Barriers identified include:   - Incentives of philanthropy don’t encourage players to openly share knowledge or even seek it in the ﬁrst place. - Philanthropy’s structure—fragmented, highly decentralized, hardly standardized—makes it difﬁcult terrain for circulating new information. - Limited professionalization and a low supply of practice standards rob philanthropy of some avenues used by other industries to share what works. - Some practitioners might seek to share their knowledge, but the majority are far less likely to use the knowledge of others. As the cause, some cite an undercurrent of competitiveness, a bias against ideas or practices “not invented here,” especially—and ironically—among foundations. - Collective amnesia of philanthropic organisations  Non-profits don’t want to communicate bad news or failure. |
| 4. | Fidelity  2017  USA | Overcoming Barriers to Giving | Study seeking to understand what holds donors back from giving more to charity and whether there are ways to overcome the barriers they face. | Quantitative study utilizing a survey. | 3254 participants who give to charity and itemize charitable deductions on their tax return. | - 65% would give more if they knew the impact of their donations. - More than 80% of respondents had concerns about how their donation will be used and if it will make a difference. - 21% revealed that they were not sure who to seek advice from. - 17% revealed that they cared about certain causes but didn’t know where to donate. |
| 5. | Jones, T  Dewling, C.  Alexander, J.  2018  Published in UK  Study conducted in Dubai, Ecuador, India, Malaysia, Singapore, UK and USA | Future of Philanthropy Insights from Multiple Expert Discussions Around the World | Study drawing on workshops and ‘conversations’ with expert participants. The study provides an overview of the collective debate and, where useful, includes references to other areas of research. | Qualitative study utilising in person discussions and 9 workshops which together had more than 200 participants. | 200 + participants in workshops drawn from academia, business, government, advisory, charities and nongovernmental organisations (NGOs) | - One driver of change is knowledge and its practical application. A common complaint from donors is the lack of understanding around the real impact of a particular donation: - Challenges include lack of resources:   – there aren’t enough people with the necessary skills to be able do the analysis. In addition, issues such as difficulties of attribution or the time-lag between intervention and results further complicate the matter.   - Donors can sometimes lack the information they need to understand the impact of their donation and to make informed decisions around future giving. - Data driven philanthropy: Often enabled by digital technology, donors are increasingly able to follow their money, see change, and directly link results to their donation. Greater transparency enables more focus on areas that can make the most difference. However, the increasing dependency on data and the widespread sharing of personal information presents risks around privacy and freedom of expression. It also means that areas of need that are not ‘measurable’ may well be neglected. - Although emotion may drive the initial choice of cause, for most donors, particularly those with a business background, some sort of quantified metrics, however sketchy, will be increasingly welcome when it comes to tracking the direction and impact of the gift. |
| 6. | Kail, A. Johnson, S. & Bowcock, M.  2016  UK | Giving more and better – How can the philanthropy sector improve? | Report is a summary of findings from a project funded by the Hazelhurst Trust and managed by NPC (New Philanthropy Capital). Examines how philanthropists are influenced and encouraged or discouraged in their giving. | Qualitative Study incorporating 9 interviews and workshops. | 9 participants largely philanthropy professionals and one academic | - Insufficient information concerning the effectiveness or otherwise of charities. - Good quality information upon which to base decisions is a pre-requisite to better quality giving. |
| 7. | Ravenscroft, C.  2013  UK | The Secrets of Success? How charitable funders use and share evidence in practice. | Discussion paper exploring how a small group of UK funders use and share evidence in practice. What evidence they draw on, how they find and use it. And how they share evidence to inform the future decisions of others – funders, practitioners, policymakers. | Qualitative study utilising semi-structured interviews. | Semi-structured interviews with 8 participants  Consultations with 5 participants | - Funders draw on a wide range of evidence sources. - The evidence funders rely on differs from the evidence they generate. - Funders are keen to know and share what works but may have limited knowledge about the relative impact and cost effectiveness of different interventions. - Grantees may be nervous about sharing evidence with funders and their evidence can be of variable quality – funders need mechanisms to help address this. - Funders could make better use of the evidence they do hold by sharing it more widely. |
| 8. | Tillotson, C.  2016  UK | Learning to give: lessons for advisers and would-be philanthropists | Study exploring why the UK’s HNWI’s and UHNWI’s give so little in the context of their overall wealth. | Mixed methods study | Sampled 500 of UK based HNWI’s also sampled 383 professional services firms offering philanthropy advice. Interviews with 22 professional participants working in philanthropy. | - On average the UK’s wealthy population gives a score of just 5.9 out of 10 for the philanthropy advice experience they receive from their professional advisers. |
| 9. | Van Poortvliet, et al.  2011  UK | Foundations for Knowledge: Sharing knowledge to increase impact: a guide for charitable funders. | Research explored the extent to which knowledge is shared across the third sector and also sought to highlight examples of good practice to share with funders. | Qualitative study featuring a literature review and semi-structured interviews | Semi-structured interviews with 12 UK foundations. | - The infrastructure for knowledge sharing across philanthropy is underdeveloped. - No single mechanism for sharing knowledge. |

## **Table 3. Characteristics of excluded studies**

|  | **Author, Year Published, Country** | **Name** | **Description** | **Study Design or Focus** | **Participants** | **Key Findings** |
| --- | --- | --- | --- | --- | --- | --- |
| 1. | Aksoy, B.  Krasteva, S.  2018  USA | When Does Less Information Translate into More Giving to Public Goods | Study exploring the impact of information provision on voluntary donations. | Experimental study. | 360 participants in the online experiment and 303 participated in the laboratory experiment | - Information increases average contributions of less generous groups. - Information reduced average contributions from more generous group |
| 2. | Barclays Wealth and Ledbury Research  2010  UK | Barriers to Giving A white paper in co-operation with Ledbury Research | Seeks to understand what is holding the wealthy back from giving more to charity. | In-depth quantitative survey of 500 high net worth individuals | 500 high net worth individuals participated in a survey. | - The first barrier is a lack of financial security, which is even more acute given the turbulent financial markets. - The second barrier is based on the wealthy’s values, where they may be missing one of the three key motivators:   - Familial,   - Societal or   - Religious reasons. - Concerns about how charities are run are a growing issue for the wealthy. - The current tax system and welfare policies have a complex relationship with wealthy philanthropists: they need to be supportive, without being interventionist. |
| 3. | Beddoes, D. Brodie, E. Clarke, R.  Hoong, S. C.  2012  UK | Benefits of open access to scholarly research for voluntary and charitable sector organisations. | Research examining the benefits of open access to scholarly research outputs to voluntary and community sector organisations. |  |  | - Voluntary and charitable sector has an appetite and need for scholarly research that it cannot currently satisfy - VCOs have identified a consistent set of barriers to accessing research - Existing OA routes and sources of support should be publicised widely to the VCS - Open Access repositories should be accessible to the voluntary and charitable sector - All publicly funded organisations should make their research available easily and at no cost to the VCS - Advice, support and training resources should be developed and made widely available to the sector to help it access (in the widest sense) research - Intermediaries should be engaged in a broad discussion about how they might provide additional support to the sector - Research should be done into the characteristics of effective relationships between the VCS and academic researchers/institutions. |
| 4. | Brody, S.  Koester, A.  Markovits, Z.  Phillips, J.  2016  USA | Moving the Needle: What Works Cities and the use of data and evidence | Explores how cities better leverage data and evidence to inform their decision making as it relates to improving the lives of their residents. | Quantitative study utilising survey which track 152 indicators scored on a 5-point Likert scale | Data collected from an initial 28 cities plus a second group of 39 cities with populations of between 100,000 and 1,000,000 | - Found that city leaders wanted to foster innovation and solve problems by utilising data but that their cities lacked the policies, performance management systems and organisational culture of using data and evidence to turn positive intentions into results. - Barriers to utilising such evidence included: - Lack of staff, - Lack of financial resources, - Limited knowledge and expertise, - Lack of trust in the data, - Old and incompatible systems for data collection and - Challenges in communicating the importance of this work to stakeholders. |
| 5. | Buchanan, P. 2019  US | Giving Done Right: Effective Philanthropy and Making Every Dollar Count’ Public Affairs Books | A practical guide to philanthropy | Offers intellectual frameworks, data-driven insights, tools, and practical examples to facilitate understanding of what it takes to make an effective philanthropic gift. | Buchanan has been working with givers for close to 20 years. His platform has been the [CEP](https://cep.org/), a research and consulting non-profit organization of which he is the founding president. CEP provides data and insights to what it calls “philanthropic funders” with the goal of increasing their effectiveness and impact | - There is a moral imperative to do the most possible good with the resources with which givers are entrusted (p. 50). - Particularly for philanthropists who have benefited from a tax break for their gift. For when the treasury subsidises philanthropic giving its aim is to create a public benefit through private giving and hence the philanthropists have a moral responsibility to give responsibly and well. |
| 6. | Butera, L. Houser, D.  2017  USA | Delegating Altruism: Towards an understanding of agency in charitable giving | Explores the economics of agency in the context of giving decisions. In particular, how competing agency and information costs determine donors’ selection into delegated giving, and how this affects levels of giving. | Laboratory experiment |  | - That agency plays a small role in the promotion of giving. - Donors do not reduce donations when algorithms guarantee efficient recipients but limit the donor’s control over recipients. - Giving circles or giving groups appeal to donors who would otherwise not make informed decisions. |
| 7. | Fiennes, C.  2012  UK | ‘It Ain’t What You Give, It’s the Way that You Give It’– Making charitable donations which get results’ – Giving Evidence | Explores how best to ensure donors secure the greatest social benefit from the resources they channel through charities to effect social change. | Not applicable. |  | - Identifies common practices of donors that cause problems for charities. - Not all charities are equally good, and their performance can vary widely. - Donors’ resources should flow in large part to the *best*performers. |
| 8. | Hardwick. R.  Anderson, R.  Cooper, C.  2015  UK | How do third sector organisations use research and other knowledge - A systematic scoping review. | Systematic search of electronic databases carried out. Literature then narratively summarised to describe how TSOs use knowledge in their decision making. | Scoping review exploring what is known about how health and social care TSOs use research in their work. |  | - Retrieved ten qualitative and mixed methods studies. - TSOs seek to make ‘evidence-based’ decisions. - Organisational context influences their preferences for particular kinds of research and knowledge and how they utilise it. - Barriers to research use include time, staff skill, resources and the contextual nature of some academic research. - Facilitators of evidence use include:   using research intermediaries and involving TSOs in research. |
| 9. | Kassatly, A.  2018 | How philanthropy infrastructure can promote evidenced-based giving | Explores why smaller grant-making organisations have yet to fully embrace data-driven philanthropy, what the potential benefits of data are and how it should be used, and how philanthropy infrastructure organisations could help. | Not applicable |  | There are a number of challenges that prevent this evidence-based approach from really taking off among smaller grant-making institutions.   - The first is capacity. Many smaller foundations do not have the capacity to do extensive analysis of impact reports on different interventions, nor do they have the in-house expertise or funding to support charities in collecting monitoring and evaluation (M&E) data - The second is motivation. Many philanthropists and smaller private foundations are more inclined to fund causes or organisations that they feel a personal connection to, not because evidence suggests they are effective - The third is focus area. A common refrain is that problems that are complex, address the future, or involve multiple stakeholders are nearly impossible to collect accurate monitoring and evaluation data or prepare comprehensive impact assessments about. - The fourth challenge is availability of data. Good quality data on successful interventions is still not widely published. - The fifth is ease. Collecting data on social issues is difficult. When the outcomes that charities are trying to affect are so varied, room for standardisation on metrics and on what data to collect is minimal. Further, to date, there has been more of a focus on outputs, which are easier to measure, though less explicit about change achieved than outcomes.   Evidence-based philanthropy is more talked about than done. Philanthropy support organisations can help to remedy this. |
| 10. | Oliver, K. et al., 2014  UK | A systematic review of barriers to and facilitators of the use of evidence by policymakers’ | Systematic search of electronic databases carried out. | Systematic search of electronic databases carried out. Studies included if they were primary research or systematic reviews about factors affecting the use of evidence in policy. |  | - Most frequently reported barriers to evidence were:   - Poor access to good quality and relevant research evidence and   - Lack of timely research output. - Most frequently reported facilitators were:   - Collaborations between researchers and policy makers and relationships and   - Improved relationships and skills |
|  | Schorr, L. B. and Farrow, F.  2011  USA | Expanding the evidence universe: doing better by knowing more | Paper seeking to address issues and provide recommendations with a view to expanding the knowledge base necessary to improve outcomes for children, families and communities. | Not applicable |  | The boundaries which the prevailing framework draws around acceptable evidence too greatly limit the knowledge base available to policy makers, program designers, and evaluators. |
| 11. | Steer, L., & Baudienville, G.  2010  UK | What drives donor financing of basic education? Overseas Development Institute. Project Briefing 39. | Explores why, despite the rhetoric of political support for EFA, the sector has not attracted the necessary funding and suggests ways in which external support for basic education could be increased. | Large number of semi-structured interviews with donor agencies and NGOs and case studies on two countries.  Draws on the findings of a recent research study commissioned by the William and Flora Hewlett Foundation. |  | - Six key factors appear to influence donor decisions around financing basic education. - The most significant relate to donor prioritisation and leadership, evidence and advocacy and aid architecture, followed by the absorptive capacity of partners, partner demand and donor capacity. |
| 12. | US Trust  2018  USA | The 2018 US Trust Study of High Net Worth Philanthropy Portraits of Generosity | The 2018 U.S. Trust Study of High Net Worth Philanthropy (the "Study") is the seventh in a biennial series of reports on the giving and volunteering practices of wealthy households in the United States. The Study is an authoritative source of information on wealthy Americans’ philanthropic attitudes and practices. | Based on a nationally representative random sample of wealthy households.  The wealth threshold for inclusion in the Study is a widely recognized standard based on the qualifying level for certain types of financial investments: an annual household income greater than $200,000 and/or net worth greater than $1,000,000 (excluding the value of the primary residence. | The total Study population in 2018 comprised 1,646 households. Forty-nine percent of respondents identified themselves as men, while 51% identified themselves as women. | - Giving is being shaped by a diverse donor universe of different ages, ethnic backgrounds and gender identities. - Women are at the forefront of philanthropic engagement and impact. - An opportunity for non-profits and advisors is highlighted by the fact that only 49% of donors have a strategy for their giving. |
| 13. | Wallace, J. Nwosu, B. and Clarke, M.  2012  UK | Barriers to the uptake of evidence from systematic reviews and meta-analyses: a systematic review of decision makers’ perceptions | A systematic review of the barriers to the uptake of evidence from systematic reviews and meta-analyses | The focus of the study was to identify barriers to the uptake of evidence from systematic reviews | 27 unique published studies included n=10218 of which 64% physicians. | The most commonly investigated barriers were lack of use (14/25), lack of awareness (12/25), lack of access (11/25), lack of familiarity (7/25), lack of usefulness (7/25), lack of motivation (4/25) and external barriers (5/25) |
