# Supplementary material for: A systematic review of the barriers to and facilitators of the use of evidence by philanthropists when determining which charities (including health charities or programmes) to fund
Source: Syst Rev. 2020 Aug 27;9:199. doi: 10.1186/s13643-020-01448-w (PMC7453541; doi:10.1186/s13643-020-01448-w)
Supplement: Supplementary file 2 — Additional file 2. GRADE CERQual Tables. [file 13643_2020_1448_MOESM2_ESM.docx]

## **Table 4: Barriers to the Use of Evidence**

| BARRIERS TO THE USE OF EVIDENCE | | | | | | | |
| --- | --- | --- | --- | --- | --- | --- | --- |
| **Review Finding 1: Philanthropists and third sector professionals encounter difficulties in accessing evidence to inform their decision making (8/9 studies)** | | | | | | | |
| STUDY | METHODS OF DATA COLLECTION | METHODS OF DATA ANALYSIS | METHODOLOGICAL LIMITATIONS | | | | |
|  |  |  | Was ethics approval granted? | Is there an appropriate description of researcher reflexivity? | Is the sampling method appropriate? | Is the method of data collection appropriate? | Is the method of data analysis appropriate? |
| **Study 1**  **2010** | Semi-structured interviews conducted over the telephone. Participants were recruited by the Charities Aid Foundation (CAF). CAF randomly selected 60 charity account holders from its database, filtering by postcode as a method of selecting approximately equal numbers of high-, medium- and lower-income donors. | The full transcripts of all 60 interviews were analysed inductively and out of this process 12 key themes emerged, the data was coded to these 12 themes.  Efforts were undertaken to ensure the reliability of coding decisions through consultations with academic colleagues with expertise in qualitative methods. | Unclear (not reported) | Not described in the study. | Yes | Yes | Yes |
| **Study 2**  **2009** | Mixed methods study combined qualitative methods utilising semi-structured interviews with a quantitative audit. The latter was intended to draw on published and web-based material to provide a basic ‘map’ of what philanthropy research and training is currently being undertaken within Europe.  Web search and desk top data collection.  The author ‘spoke’ with 40 participants | Bridget Pettitt (BP Research Consultancy) assisted the data gathering and analysis by collating the information provided to us and web-searching for examples of universities and other organisations involved in philanthropy research and teaching.  The material collected for the quantitative audit was used to inform the report and its conclusions but the data – without further verification –was offered to the European Foundation Centre (EFC) and to the European Research Network on Philanthropy (ERNOP) | Unclear (not reported) | Not described in study but the report was commissioned with the support of The Adessium Foundation, The Atlantic Philanthropies and The Pears Foundation..  The Network of European Foundations (NEF) administered the funding of the work on the study. | Yes | No | Unclear (not clearly described) |
| **Study 3**  **2003** | Qualitative study in which 12 individuals working within philanthropy were interviewed – 10 participant organisations also contributed to the study. | Unclear (not reported) | Unclear (not reported) | Not described in the study. | Yes | Yes | Unclear (not described) |
| **Study 4**  **2017** | Quantitative study seeking to understand what holds donors back from giving more to charity and whether there are ways to overcome the barriers they face. | Report is based on a 2016 survey conducted among 3254 participants in the US who give to charity and itemise charitable deductions on their tax return. The study was conducted by Artemis Strategy Group an independent research firm. | Unclear (not reported) | Not described in the study. | Yes | Yes | Unclear (not clearly described) |
| **Study 5**  **2018** | A qualitative study drawing on workshops and ‘conversations’ with expert participants. The study provides an overview of the collective debate and, where useful, includes references to other areas of research. | The authors of the report conducted a number of workshops across seven countries with more than 200 participant ‘experts’. It is unclear what qualifies someone as an expert. | Unclear (not reported) | Not described in the study. | Yes | Yes | Unclear (not clearly described) |
| **Study 6**  **2016** | Qualitative study incorporating 9 interviews and workshops. | Unclear (not reported) | Unclear (not reported) | Not described in the study. | Yes | Yes | Unclear (not clearly described) |
| **Study 7**  **2013** | Semi-structured interviews with 8 participants.  Consultations with 5 participants | Unclear (not reported) | Unclear (not reported) | Moderate amount of reflexivity. The author commented that she came to the project ‘*with some starting assumptions, based on my previous experiences with funding. Among my inspirations is a motto of Give Well, a US donor advisory service: “Information about how to help people should never be secret”. I am confident that funders would agree with this principle – the real questions are about how this is done effectively, and sensitively, in practice*.’  The study was developed on behalf of the Alliance for Useful Evidence and was discussed by funders at the Intelligent Funding Forum in May 2013 | Yes | Yes | Unclear (not clearly described) |
| **Study 9**  **2011** | Qualitative study featuring a literature review and semi-structured interviews. | Unclear (not reported) | Unclear (not reported) | Not described in the study. | Unclear (not reported) | Yes | Unclear (not reported) |

| BARRIERS TO THE USE OF EVIDENCE | | | | | | | |
| --- | --- | --- | --- | --- | --- | --- | --- |
| **Review Finding 2: Philanthropists and third sector professionals experience challenges in understanding the evidence (5/9 studies)** | | | | | | | |
| **STUDY** | METHODS OF DATA COLLECTION | METHODS OF DATA ANALYSIS | METHODOLOGICAL LIMITATIONS | | | | |
|  |  |  | Was ethics approval granted? | Is there an appropriate description of researcher reflexivity? | Is the sampling method appropriate? | Is the method of data collection appropriate? | Is the method of data analysis appropriate? |
| **Study 1**  **2010** | Semi-structured interviews conducted over the telephone. Participants were recruited by the Charities Aid Foundation (CAF). CAF randomly selected 60 charity account holders from its database, filtering by postcode as a method of selecting approximately equal numbers of high-, medium- and lower-income donors. | The full transcripts of all 60 interviews were analysed inductively and out of this process 12 key themes emerged.  The data were coded to these 12 themes.  Efforts were undertaken to ensure the reliability of coding decisions through consultations with academic colleagues with expertise in qualitative methods. | Unclear (not reported) | Not described in the study. | Yes | Yes | Yes |
| **Study 2**  **2009** | Mixed methods study combined qualitative methods utilising semi-structured interviews with a quantitative audit. The latter was intended to draw on published and web-based material to provide a basic ‘map’ of what philanthropy research and training is currently being undertaken within Europe.  Web search and desk top data collection.  The author ‘spoke’ with 40 participants | Bridget Pettitt (BP Research Consultancy) assisted the data gathering and analysis by collating the information provided to us and web-searching for examples of universities and other organisations involved in philanthropy research and teaching.  The material collected for the quantitative audit was used to inform the report and its conclusions but the data – without further verification –was offered to the European Foundation Centre (EFC) and to the European Research Network on Philanthropy (ERNOP) | Unclear (not reported) | Not described in study but the report was commissioned with the support of The Adessium Foundation, The Atlantic Philanthropies and The Pears Foundation.  The Network of European Foundations (NEF) administered the funding of the work on the study. | Yes | No | Unclear (not clearly described) |
| **Study 4**  **2017** | Qualitative study in which 12 individuals working within philanthropy were interviewed – 10 participant organisations also contributed to the study. | Report is based on a 2016 survey conducted among 3254 participants in the US who give to charity and itemise charitable deductions on their tax return. The study was conducted by Artemis Strategy Group an independent research firm. | Unclear (not reported) | Unclear (not described in the study). | Yes | Yes | Unclear (not clearly described) |
| **Study 5**  **2018** | Quantitative study seeking to understand what holds donors back from giving more to charity and whether there are ways to overcome the barriers they face. | Unclear (not reported) | Unclear (not reported) | Unclear (not described in the study). | Yes | Yes | Unclear (not clearly described) |
| **Study 7**  **2013** | Semi-structured interviews with 8 participants.  Consultations with 5 participants | Unclear (not reported) | Unclear (not reported) | Moderate amount of reflexivity. The author commented that she came to the project ‘*with some starting assumptions, based on my previous experiences with funding. Among my inspirations is a motto of Give Well, a US donor advisory service: “Information about how to help people should never be secret”. I am confident that funders would agree with this principle – the real questions are about how this is done effectively, and sensitively, in practice*.’  The study was developed on behalf of the Alliance for Useful Evidence and was discussed by funders at the Intelligent Funding Forum in May 2013 | Yes | Yes | Unclear (not clearly described) |
| **Study 9**  **2011** | Qualitative study featuring a literature review and semi-structured interviews. | Unclear (not reported) | Unclear (not reported) | Not described in the study. | Unclear (not reported) | Yes | Unclear (not reported) |
| BARRIERS TO THE USE OF EVIDENCE | | | | | | | |
| **Review Finding 3: Philanthropists and third sector professionals have insufficient resources to utilise evidence (6/9 studies)** | | | | | | | |
| **STUDY** | METHODS OF DATA COLLECTION | METHODS OF DATA ANALYSIS | METHODOLOGICAL LIMITATIONS | | | | |
|  |  |  | Was ethics approval granted? | Is there an appropriate description of researcher reflexivity? | Is the sampling method appropriate? | Is the method of data collection appropriate? | Is the method of data analysis appropriate? |
| **Study 1**  **2010** | Semi-structured interviews conducted over the telephone. Participants were recruited by the Charities Aid Foundation (CAF). CAF randomly selected 60 charity account holders from its database, filtering by postcode as a method of selecting approximately equal numbers of high-, medium- and lower-income donors. | The full transcripts of all 60 interviews were analysed inductively, and out of this process 12 key themes emerged.  The data were coded to these 12 themes.  Efforts were undertaken to ensure the reliability of coding decisions through consultations with academic colleagues with expertise in qualitative methods. | Unclear (not reported) | Not described in study. | Yes | Yes | Yes |
| **Study .2.**  **2009** | Mixed methods study combined qualitative methods utilising semi-structured interviews with a quantitative audit. The latter was intended to draw on published and web-based material to provide a basic ‘map’ of what philanthropy research and training is currently being undertaken within Europe.  Web search and desk top data collection.  The author ‘spoke’ with 40 participants | Bridget Pettitt (BP Research Consultancy) assisted the data gathering and analysis by collating the information provided to us and web-searching for examples of universities and other organisations involved in philanthropy research and teaching.  The material collected for the quantitative audit was used to inform the report and its conclusions but the data – without further verification –was offered to the European Foundation Centre (EFC) and to the European Research Network on Philanthropy (ERNOP) | Unclear (not reported) | Not described in study but the report was commissioned with the support of The Adessium Foundation, The Atlantic Philanthropies and The Pears Foundation  The Network of European Foundations (NEF) administered the funding of the work on the study. | Yes | No | Unclear (not clearly described) |
| **Study 5**  **2018** | A qualitative study drawing on workshops and ‘conversations’ with expert participants. The study provides an overview of the collective debate and, where useful, includes references to other areas of research. | Unclear (not reported) | Unclear (not reported) | Not described in the study. | Yes | Yes | Unclear (not clearly described) |
| **Study 7**  **2013** | Semi-structured interviews with 8 participants.  Consultations with 5 participants | Unclear (not reported) | Unclear (not reported) | Moderate amount of reflexivity. The author commented that she came to the project ‘*with some starting assumptions, based on my previous experiences with funding. Among my inspirations is a motto of Give Well, a US donor advisory service: “Information about how to help people should never be secret”. I am confident that funders would agree with this principle – the real questions are about how this is done effectively, and sensitively, in practice*.’  The study was developed on behalf of the Alliance for Useful Evidence and was discussed by funders at the Intelligent Funding Forum in May 2013 | Yes | Yes | Unclear (not clearly described) |
| **Study 8**  **2016** | Mixed methods study exploring why the UK’s HNWIs and UHNWIs give so little in the context of their overall wealth. | Sampled 503 UK based HNWIs and also sampled 383 professional services firms offering philanthropy advice. Interviews with 22 professional participants working in philanthropy. |  | Not described in the study. |  |  |  |
| **Study 9**  **2011** | Qualitative study featuring a literature review and semi-structured interviews. | Unclear (not reported) | Unclear (not reported) | Not described in the study. | Unclear (not reported) | Yes | Unclear (not reported) |

## **Table 5: Facilitators of the Use of Evidence**

| FACILITATORS OF THE USE OF EVIDENCE | | | | | | | |
| --- | --- | --- | --- | --- | --- | --- | --- |
| **Review Finding 4: Knowledge transfer and ease of access facilitates the use of evidence (6/9 studies)** | | | | | | | |
| **STUDY** | METHODS OF DATA COLLECTION | METHODS OF DATA ANALYSIS | METHODOLOGICAL LIMITATIONS | | | | |
|  |  |  | Was ethics approval granted? | Is there an appropriate description of researcher reflexivity? | Is the sampling method appropriate? | Is the method of data collection appropriate? | Is the method of data analysis appropriate? |
| **Study 2**  **2009** | Mixed methods study combined qualitative methods utilising semi-structured interviews with a quantitative audit. The latter was intended to draw on published and web-based material to provide a basic ‘map’ of what philanthropy research and training is currently being undertaken within Europe.  Web search and desk top data collection.  The author ‘spoke’ with 40 participants | Bridget Pettitt (BP Research Consultancy) assisted the data gathering and analysis by collating the information provided to us and web-searching for examples of universities and other organisations involved in philanthropy research and teaching.  The material collected for the quantitative audit was used to inform the report and its conclusions but the data – without further verification –was offered to the European Foundation Centre (EFC) and to the European Research Network on Philanthropy (ERNOP) | Unclear (not reported) | Not described in study but the report was commissioned with the support of The Adessium Foundation, The Atlantic Philanthropies and The Pears Foundation.  The Network of European Foundations (NEF) administered the funding of the work on the study. | Yes | No | Unclear (not clearly described) |
| **Study 3**  **2003** | A qualitative study in which 12 individuals working in philanthropy were interviewed. | Unclear (not reported) | Unclear (not reported) |  | Yes | Yes | Unclear (not clearly described) |
| **Study 5**  **2018** | A qualitative study drawing on workshops and ‘conversations’ with expert participants. The study provides an overview of the collective debate and, where useful, includes references to other areas of research. | Unclear (not reported) | Unclear (not reported) | Not described in the study. | Yes | Yes | Unclear (not clearly described) |
| **Study 6**  **2016** | Expert interviews with 9 participants.  Workshops informed the project and key organisations dedicated to developing greater philanthropy in the UK participated (NPC, The Philanthropy Workshop, Philanthropy Impact, Beacon Awards for Philanthropy, and Ten Years’ Time) | Unclear (not reported) | Unclear (not reported) | Not described in the study. | Yes | Unclear (not described) | Unclear (not clearly described) |
| **Study 7**  **2013** | Semi-structured interviews with 8 participants.  Consultations with 5 participants | Unclear (not reported) | Unclear (not reported) | Moderate amount of reflexivity. The author commented that she came to the project ‘*with some starting assumptions, based on my previous experiences with funding. Among my inspirations is a motto of Give Well, a US donor advisory service: “Information about how to help people should never be secret”. I am confident that funders would agree with this principle – the real questions are about how this is done effectively, and sensitively, in practice*.’  The study was developed on behalf of the Alliance for Useful Evidence and was discussed by funders at the Intelligent Funding Forum in May 2013 | Yes | Yes | Unclear (not clearly described) |
| **Study 9**  **2011** | Qualitative study featuring a literature review and semi-structured interviews. | Unclear (not reported) | Unclear (not reported) | Not described in the study. | Unclear (not reported) | Yes | Unclear (not reported) |
| FACILITATORS OF THE USE OF EVIDENCE | | | | | | | |
| **Review Finding 5: Professional advisors facilitate the uptake of evidence (4/9 studies)** | | | | | | | |
| **STUDY** | METHODS OF DATA COLLECTION | METHODS OF DATA ANALYSIS | METHODOLOGICAL LIMITATIONS | | | | |
|  |  |  | Was ethics approval granted? | Is there an appropriate description of researcher reflexivity? | Is the sampling method appropriate? | Is the method of data collection appropriate? | Is the method of data analysis appropriate? |
| **Study 2**  **2009** | Mixed methods study combined qualitative methods utilising semi-structured interviews with a quantitative audit. The latter was intended to draw on published and web-based material to provide a basic ‘map’ of what philanthropy research and training is currently being undertaken within Europe.  Web search and desk top data collection.  The author ‘spoke’ with 40 participants | Bridget Pettitt (BP Research Consultancy) assisted the data gathering and analysis by collating the information provided to us and web-searching for examples of universities and other organisations involved in philanthropy research and teaching.  The material collected for the quantitative audit was used to inform the report and its conclusions but the data – without further verification –was offered to the European Foundation Centre (EFC) and to the European Research Network on Philanthropy (ERNOP) | Unclear (not reported) | Not described in study but the report was commissioned with the support of The Adessium Foundation, The Atlantic Philanthropies and The Pears Foundation.  The Network of European Foundations (NEF) administered the funding of the work on the study. | Yes | No | Unclear (not clearly described) |
| **Study 7**  **2013** | Semi-structured interviews with 8 participants.  Consultations with 5 participants | Unclear (not reported) | Unclear (not reported) | Moderate amount of reflexivity. The author commented that she came to the project ‘*with some starting assumptions, based on my previous experiences with funding. Among my inspirations is a motto of Give Well, a US donor advisory service: “Information about how to help people should never be secret”. I am confident that funders would agree with this principle – the real questions are about how this is done effectively, and sensitively, in practice*.’  The study was developed on behalf of the Alliance for Useful Evidence and was discussed by funders at the Intelligent Funding Forum in May 2013 | Yes | Yes | Unclear (not clearly described) |
| **Study 8**  **2016** | Mixed methods study examining why the UK’s HNWIs and UHNWIs give so little in the context of their overall wealth. | Directional analysis of the data. | Not required | Yes, a commercial relationship existed between Scorpio and Philanthropy Impact. | Yes | Yes | Yes |
| **Study 9**  **2011** | Qualitative study featuring a literature review and semi-structured interviews. | Unclear (not reported) | Unclear (not reported) | Not described in the study | Unclear (not reported) | Yes | Unclear (not reported) |
| FACILITATORS OF THE USE OF EVIDENCE | | | | | | | |
| **Review Finding 6: A broader definition of credible evidence and better standardisation of reporting will facilitate the use of evidence (4/9 studies)** | | | | | | | |
| **STUDY** | METHODS OF DATA COLLECTION | METHODS OF DATA ANALYSIS | METHODOLOGICAL LIMITATIONS | | | | |
|  |  |  | Was ethics approval granted? | Is there an appropriate description of researcher reflexivity? | Is the sampling method appropriate? | Is the method of data collection appropriate? | Is the method of data analysis appropriate? |
| **Study 2**  **2009** | Mixed methods study combined qualitative methods utilising semi-structured interviews with a quantitative audit. The latter was intended to draw on published and web-based material to provide a basic ‘map’ of what philanthropy research and training is currently being undertaken within Europe.  Web search and desk top data collection.  The author ‘spoke’ with 40 participants | Bridget Pettitt (BP Research Consultancy) assisted the data gathering and analysis by collating the information provided to us and web-searching for examples of universities and other organisations involved in philanthropy research and teaching.  The material collected for the quantitative audit was used to inform the report and its conclusions but the data – without further verification –was offered to the European Foundation Centre (EFC) and to the European Research Network on Philanthropy (ERNOP) | Unclear (not reported) | Not described in study but the report was commissioned with the support of The Adessium Foundation, The Atlantic Philanthropies and The Pears Foundation.  The Network of European Foundations (NEF) administered the funding of the work on the study. | Yes | No | Unclear (not clearly described) |
| **Study 5**  **2018** | A qualitative study drawing on workshops and ‘conversations’ with expert participants. The study provides an overview of the collective debate and, where useful, includes references to other areas of research. | Unclear (not reported) | Unclear (not reported) | Not described in the study. | Yes | Yes | Unclear (not clearly described) |
| **Study 6**  **2013** | Semi-structured interviews with 8 participants.  Consultations with 5 participants | Unclear (not reported) | Unclear (not reported) | Moderate amount of reflexivity. The author commented that she came to the project ‘*with some starting assumptions, based on my previous experiences with funding. Among my inspirations is a motto of Give Well, a US donor advisory service: “Information about how to help people should never be secret”. I am confident that funders would agree with this principle – the real questions are about how this is done effectively, and sensitively, in practice*.’  The study was developed on behalf of the Alliance for Useful Evidence and was discussed by funders at the Intelligent Funding Forum in May 2013 | Yes | Yes | Unclear (not clearly described) |
| **Study 9**  **2011** | Qualitative study featuring a literature review and semi-structured interviews. | Unclear (not reported) | Unclear (not reported) | Not described in the study however the study forms part of New Philanthropy Capital’s series of reports. They reveal that they set up an advisory group comprising six funders which provided advice and feedback during the research. | Unclear (not reported) | Yes | Unclear (not reported) |

## **Table 6: Data contributing to each of the findings:**

| BARRIERS TO THE USE OF EVIDENCE **Review Finding 1: Philanthropists and third sector professionals encounter difficulties in accessing evidence to inform their decision making (6/9 studies)** | | | | |
| --- | --- | --- | --- | --- |
| **STUDY** | DATA FROM THE INDIVIDUAL STUDIES THAT CONTRIBUTED TO THE FINDING | STUDY AIM | STUDY SETTING | STUDY PARTICIPANTS |
| **Study 1**  **2010** | ‘S*ome donors experience feelings of stress, anxiety, befuddlement and exasperation and there is widespread acknowledgement that choices are inevitably based on partial information, as the vast number of options makes it impossible to rationally assess them all’*.  Theme: Volume of information  The study refers to the difficulties in obtaining … objective information on charities’ general competence.  Theme: Lack of Evidence | Study seeks to explore the role of personal taste and experience in determining which charities donors choose to support. | UK | 60 participants who are all charity account holders with the Charities Aid Foundation (CAF). CAF randomly selected the participants from its database, filtering by postcode to ensure the sample contained approximately equal numbers of high, medium and lower income donors. Of the 60 interviewees, 22 were women and 38 were men; a third were from the north and two‐thirds from the south. There was a spread of ages from people in their thirties to their eighties, with people in their sixties being the most highly represented. |
| **Study 2**  **2009** | There is a deficiency of knowledge within the philanthropic sector about sources of knowledge or evidence.  Theme: Lack of Awareness of Evidence Sources  The study identified insufficient commitment to knowledge transfer as a barrier to the use of evidence with neither the philanthropy sector nor academics ‘*sufficiently committed throughout their practice to knowledge transfer – “it’s an add-on … there’s no real investment in it…”’.*  Theme: Knowledge transfer | Study explores the availability and use within Europe of research into philanthropy and social investment and how a stronger and more effective framework could be built to enhance and extend opportunities for study and for learning in order to improve the practice of philanthropy. | Europe and UK | 40 participants engaged in work to improve the scale, quality and depth of research and learning. |
| **Study 3**  **2003** | The typical dissemination strategy … stumbles by implicitly assuming that learning and change happen once information is broadly transmitted and made available…. Rather than spread knowledge a mile wide and an inch deep, R&D organisations would do well to target the desired practitioner actions that their knowledge inform.  Theme: Difficulties in dissemination  The majority of practitioners are far less likely ‘*to use the knowledge of others … some cite an undercurrent of competitiveness, a bias against ideas or practices “not invented here”’*  Theme: Competitiveness | Explores how good ideas are circulated within philanthropy. | USA | The researchers (Williams Group) interviewed a dozen individuals who are regarded as opinion leaders and information gatekeepers in the field. Williams group have interspersed the observations of participants throughout the paper and also drew on their own work over the previous five years in which they helped philanthropic organisations to develop communications programs. The paper was also informed by the emerging work of Williams Group with each of the participant groups. |
| **Study 5**  **2018** | Study reveals that donors sometimes lack the information that they need to ‘*understand the impact of their donations’.*  Theme: Lack of Evidence  Study also identified a number of challenges relating to measurement ‘*measuring everything from protecting the environment to tackling world hunger on the same terms is tricky’.* Consequently, *‘Measuring the benefits of philanthropy is surprisingly hard’.*  Theme: Measurement  Moreover, all too frequently the wrong thing is measured – for example measuring activities rather than outcomes.  Theme: Measurement | Qualitative study which seeks to capture an overview of the collective debate on the future of philanthropy. | Dubai, Ecuador, India, Malaysia, Singapore, UK and USA, | 200 + participants drawn from academia, business, government, advisory, charities and NGOs. |
| **Study 7**  **2013** | Study found that some charity grantees can struggle to provide the evidence sought by the funder. This is particularly true of smaller charities which often struggle to provide even basic output data. *‘Applicants find it difficult to provide the right sort of evidence for us.’*  Theme: Lack of Evidence  Also revealed that boards of grant-making foundations need a bespoke synthesis of evidence but that this is rarely provided in the shape they need.  Theme: Availability  Short term funding doesn’t generate evidence about what doesn’t work.  Theme: Fear of Failure  The study identified insufficient knowledge transfer as a barrier to accessing evidence. ‘*Funders could make better use of the evidence they do hold by sharing it more widely’.*  Theme: Knowledge transfer | Discussion paper exploring how a small group of UK funders use and share evidence in practice. What evidence they draw on, how they find and use it. And how they share evidence to inform the future decisions of others – funders, practitioners, policymakers. | UK | Focus of the study is on the experiences of grant-making trusts and foundations. |
| Study 9  2011 | Study revealed that lessons revealing what doesn’t work are rarely publicised.  Theme: Fear of Failure  The study also acknowledged that there knowledge sharing was not working as well as many would like.  Theme: Knowledge transfer | This study explored the extent to which knowledge is shared across the third sector and also sought to highlight examples of good practice to share with funders. | UK | 12 participants from UK foundations. |

| BARRIERS TO THE USE OF EVIDENCE **Review Finding 2: Philanthropists and third sector professionals encounter challenges in understanding the evidence (3/9studies)** | | | | |
| --- | --- | --- | --- | --- |
| **STUDY** | DATA FROM THE INDIVIDUAL STUDIES THAT CONTRIBUTED TO THE FINDING | STUDY AIM | STUDY SETTING | STUDY PARTICIPANTS |
| **Study 1**  **2010** | *‘Unless you pour over books and understand what you’re reading, I think it’s very difficult’ (Female 70s middle income).*  *‘Given the difficulties in obtaining (and understanding) objective information om charities’ general competence.’*  Theme: Constraints around understanding | Study seeks to explore the role of personal taste and experience in determining which charities donors choose to support. | UK | 60 participants who are all charity account holders with the Charities Aid Foundation (CAF). CAF randomly selected the participants from its database, filtering by postcode to ensure the sample contained approximately equal numbers of high, medium and lower income donors. Of the 60 interviewees, 22 were women and 38 were men; a third were from the north and two‐thirds from the south. There was a spread of ages from people in their thirties to their eighties, with people in their sixties being the most highly represented. |
| **Study 5**  **2018** | Study revealed that it a common complaint from donors related to an inability to understand the impact of their donation.  ‘*We give away a lot of money, but we don’t think that we do it very well’.*  Theme: Constraints around understanding | Qualitative study which seeks to capture an overview of the collective debate on the future of philanthropy. | Dubai, Ecuador, India, Malaysia, Singapore, UK and USA, | 200 + participants drawn from academia, business, government, advisory, charities and NGOs. |
| **Study 7**  **2013** | This study identified a lack of synthesized evidence as a challenge for funders: ‘*The Board need a bespoke synthesis of evidence – it’s easy to go foraging around for information, but this rarely comes in the shape they need.’*  Theme: Synthesized evidence  *‘I would say that we sit on a whole bunch of data but I couldn’t tell you what works and what doesn’t’*  Theme: Synthesized evidence    *‘A further challenge for funders using evidence at a strategic level is synthesising information…*  Theme: Synthesized evidence | Discussion paper exploring how a small group of UK funders use and share evidence in practice. What evidence they draw on, how they find and use it. And how they share evidence to inform the future decisions of others – funders, practitioners, policymakers. | UK | Focus of the study is on the experiences of grant-making trusts and foundations. |

| BARRIERS TO THE USE OF EVIDENCE**Review Finding 3: Insufficient resources make it harder to access and understand evidence (6/9 studies)** | | | | |
| --- | --- | --- | --- | --- |
| **STUDY YEAR** | DATA FROM THE INDIVIDUAL STUDIES THAT CONTRIBUTED TO THE FINDING | STUDY AIM | STUDY SETTING | STUDY PARTICIPANTS |
| **Study 1**  **2010** | Study revealed that many donors felt overwhelmed by the quantity of information they received from charities: *‘…. You are bombarded. It’s amazing what comes through the door and you’ve got no means of making an objective judgment.’*  Theme: Volume of information  The study referred to the possibility that some people do not give because ‘*they are overwhelmed by choice and are experiencing “analysis paralysis”’.*  Theme: Volume of information | Study seeks to explore the role of personal taste and experience in determining which charities donors choose to support. | UK | 60 participants who are all charity account holders with the Charities Aid Foundation (CAF). CAF randomly selected the participants from its database, filtering by postcode to ensure the sample contained approximately equal numbers of high, medium and lower income donors. Of the 60 interviewees, 22 were women and 38 were men; a third were from the north and two‐thirds from the south. There was a spread of ages from people in their thirties to their eighties, with people in their sixties being the most highly represented. |
| **Study 2**  **2009** | Perception that the philanthropy sector is uninterested in and unwilling to pay for research into their own behaviour, effectiveness or impact;  Theme: Cost  Philanthropic resources are limited – never sufficient to meet all the demands that may be made of them.  Theme: Insufficient resources  Further perception that practitioners to perceive academics as being prone to the ‘over collection’ of data and inclined to be dismissive of many of the questions that practitioners would like studied.  Theme: Volume of information | Study explores the availability and use  within Europe of research into philanthropy and social investment and how a stronger and more effective framework could be built to enhance and extend opportunities for study and for learning in order to improve the practice of philanthropy. | Europe and the UK | 40 participants engaged in work to improve the scale, quality and depth of research and learning. |
| **Study 5**  **2018** | This study acknowledged that many do not have sufficient staff to collect and analyse evidence: *‘there aren’t enough people with the necessary skills to be able to do the analysis’*  Theme: Insufficient Staff  Theme: Lack of Skills  Insufficient resources was a recurrent theme in this study: *‘The situation is exacerbated by lack of capacity. There are some very good smaller organisations that simply do not have the technology to gather data or the staff with the skills to interpret the results.’*  Theme: Insufficient resources  Theme: Lack of Skills | Qualitative study which seeks to capture an overview of the collective debate on the future of philanthropy. | Dubai, Ecuador, India, Malaysia, Singapore, UK and USA, | 200 + participants drawn from academia, business, government, advisory, charities and NGOs. |
| **Study 7**  **2013** | This study acknowledged that funding constraints play a role in limited access to evidence: ‘*When money is tight, how will organisations survive if they have to spend a fortune on evaluations?”*  Theme: Cost  *‘In common with other funders interviewed for this report…’* Big ‘*lack the evidence or expertise to assess the relative impact and cost-effectiveness of different interventions’.*  Theme: Lack of Skills | Discussion paper exploring how a small group of UK funders use and share evidence in practice. What evidence they draw on, how they find and use it. And how they share evidence to inform the future decisions of others – funders, practitioners, policymakers. | UK | Focus of the study is on the experiences of grant-making trusts and foundations. |
| **Study 8**  **2016** | *‘One of the reasons that the UKs wealthy population hasn’t fully learned how to give is because advisors are not yet joining up the expertise needed to help would be donors give more effectively’*  Theme: Expert Opinion  Theme: Knowledge Sharing | Study exploring why the UK’s HNWIs and UHNWIs give so little in the context of their overall wealth. | UK | 503 participants drawn from UK all HNWIs also sampled 383 professional services firms and interviewed 22 people working in the philanthropy sector. |
| **Study 9**  **2011** | This study acknowledged that there was a lack of investment in learning and knowledge sharing.  Theme: Cost | This study explored the extent to which knowledge is shared across the third sector and also sought to highlight examples of good practice to share with funders. | UK | 12 participants from UK foundations |

| FACILITATORS OF THE USE OF EVIDENCE **Review Finding 4: Knowledge sharing and ease of access facilitates the use of evidence (6/9 studies)** | | | | |
| --- | --- | --- | --- | --- |
| **STUDY** | DATA FROM THE INDIVIDUAL STUDIES THAT CONTRIBUTED TO THE FINDING | STUDY AIM | STUDY SETTING | STUDY PARTICIPANTS |
| **Study 2**  **2009** | The study found that when infrastructures for knowledge sharing exist they facilitate not only:  *‘peer-to-peer sharing of experience and practice but also provide an opportunity for practitioners to hear about and engage with research findings as part of the learning experience’.*  Theme: Knowledge Sharing  Theme: Peer to Peer  Capacity building is another mechanism by which the use of evidence can be facilitated: In this way ‘*national associations of donors and other specialist networks provide comprehensive programmes of peer-to-peer capacity building, staff development and other learning opportunities … for individual philanthropists and the staff and board members of philanthropic organisations.*  Theme: Capacity Building  Theme: Knowledge Sharing  The study also identified a ‘*range of institutional and non-profit publishers of internet and paper materials’* that are providing ‘*philanthropy practitioners with a constant flow of operational guidance and factual material, dissemination and discussion of research findings, programme and project evaluations, policy debate, and links to relevant evidence and reports from beyond Europe.*’  Theme: Knowledge Dissemination  Theme: Published Materials | Study explores the availability and use within Europe of research into philanthropy and social investment and how a stronger and more effective framework could be built to enhance and extend opportunities for study and for learning in order to improve the practice of philanthropy. | Europe and UK | 40 participants engaged in work to improve the scale, quality and depth of research and learning. |
| **Study 3**  **2003** | The study finds that knowledge sharing is a ‘*long-term, ongoing process’* and that it is also critical to consider the demand for knowledge – what do knowledge users need? *‘Knowledge entrepreneurs need to get good at understanding demand for knowledge and preferred ways to access it. It wouldn’t be hard to create feedback loops to understand what’* funders and donors want and need.  Theme: Knowledge Sharing  Theme: Feedback loops  The study found that the most useful knowledge acquisition came through peer to peer networks. ‘*The people I rely om are good at connecting with others and at building a knowledge base openly. They don’t treat knowledge like copyrighted materials’.*  Theme: Peer to Peer | Explores how good ideas are circulated within philanthropy. | USA | The researchers (Williams Group) interviewed a dozen individuals who are regarded as opinion leaders and information gatekeepers in the field. Williams group have interspersed the observations of participants throughout the paper and also drew on their own work over the previous five years in which they helped philanthropic organisations to develop communications programs. The paper was also informed by the emerging work of Williams Group with each of the participant groups. |
| **Study 5**  **2018** | This study identified the application of data analytics which will play an increasingly important role in enabling people to make informed decisions. ‘…*it is clear that those who seek to maximize the impact of philanthropy … are keen on changing the current state of paucity of data, transparency and feedback loops which if optimized would ultimately result in enhanced levels of trust between all stakeholders in the philanthropy nexus.’*  Theme: Data Analytics  Theme: Feedback Loops  The report reaffirmed that: *‘Greater knowledge and understanding, together with working feedback loops were viewed as essential pre-cursors to more impactful philanthropy’.*  To this end the report recommended that the ‘*best investment a philanthropist can make is to help an organisation develop the core skills of the workers and build the knowledge systems to grow and improve the impact of the organisation.’* | Qualitative study which seeks to capture an overview of the collective debate on the future of philanthropy. | Dubai, Ecuador, India, Malaysia, Singapore, UK and USA, | 200 + participants drawn from academia, business, government, advisory, charities and NGOs. |
| **Study 6**  **2016** | This study acknowledged that access to high quality information facilitates evidence informed giving. ‘*Having good quality information to base decisions on is a necessary prerequisite to better quality of giving.’*  Theme: Ease of Access | Qualitative study seeking to explore how the philanthropy sector can improve. | UK | Nine participants drawn chiefly from the third sector – one academic. |
| **Study 7**  **2013** | The study recommends funders proactively participate in knowledge sharing and recommends that they engage with the What Works Centres ‘*both to share evidence for dissemination and to seek evidence that will inform their funding’.*  Theme: Knowledge Hubs  The study also identified professional networks as places for sharing learnings: ‘*The Association of Charitable Foundation’s issue-based networks were widely cited in interviews and generally described as ‘excellent’ and a ‘good place to come together’.’*  *‘In my experience, the Environmental Funders Network is a highly effective model. It’s funded by a number of partners, who pool resources to employ one person. They … analyse gaps in environmental funding and produce reports that are useful for all of us…’*  Theme: Networks  Information about how to help people should never be kept secret. | Discussion paper exploring how a small group of UK funders use and share evidence in practice. What evidence they draw on, how they find and use it. And how they share evidence to inform the future decisions of others – funders, practitioners, policymakers. | UK | Focus of the study is on the experiences of grant-making trusts and foundations. |
| **Study 9**  **2011** | This study points out that charitable funders are ‘*uniquely placed to learn and share knowledge’* and that by ‘*learning from their own work and that of peers, funders can make better decisions; by sharing what they know , they can create wider influence – ensuring that the best approaches are adopted by government, other funders and charities.’*  Theme: Knowledge Sharing  Theme: Peer to Peer  The study recommends that the sector as a whole creates an infrastructure with ‘centralised resources’ to facilitate knowledge sharing and learning. It suggests that the Association of Charitable Foundations is well placed to develop such an infrastructure and to host resources. Such an infrastructure will require financial support from philanthropists and foundations.  Theme: Infrastructure | This study explored the extent to which knowledge is shared across the third sector and also sought to highlight examples of good practice to share with funders. | UK | 12 participants from UK foundations. |

| FACILITATORS OF THE USE OF EVIDENCE **Review Finding 5: Professional advisors and networks facilitate the use of evidence (3/9)** | | | | |
| --- | --- | --- | --- | --- |
| **STUDY** | DATA FROM THE INDIVIDUAL STUDIES THAT CONTRIBUTED TO THE FINDING | STUDY AIM | STUDY SETTING | STUDY PARTICIPANTS |
| **Study 2**  **2009** | Identified ‘consultancies’, ‘think tanks’ and independent researchers specialising in philanthropy as facilitators of evidence as they ‘*invest extensively in the dissemination and sharing of the findings and practical application of their work – using a wide range of communication methods and systems.’*  Theme: Philanthropic Consultancies  Theme: Philanthropic Advisors | Study explores the availability and use within Europe of research into philanthropy and social investment and how a stronger and more effective framework could be built to enhance and extend opportunities for study and for learning in order to improve the practice of philanthropy. | Europe and UK | 40 participants engaged in work to improve the scale, quality and depth of research and learning. |
| **Study 7**  **2013** | Study recognised that many several funders proactively seek to involve ‘*external experts in their assessment of … funding bids. Funders considered the role of experts as complementing staff experience with technical or on the ground insights.’*  Theme: Experts | Discussion paper exploring how a small group of UK funders use and share evidence in practice. What evidence they draw on, how they find and use it. And how they share evidence to inform the future decisions of others – funders, practitioners, policymakers. | UK | Focus of the study is on the experiences of grant-making trusts and foundations. |
| **Study 8**  **2016** | This study concludes that access to high quality philanthropy advisors can facilitate better and more informed giving by philanthropists.  *‘A … telling data point shows that while only 12% of the UK’s wealthy population currently take any philanthropy advice, their giving accounts for £770 million of the total £1.3 billion: that’s 58%.’*  *‘Not knowing how to give is not entirely the fault of the UK’s wealthy, however. In the same research we mapped 380 of the UK’s 16,000 professional advisory firms and found that only 1 in 5 currently offers any kind of philanthropy advice and the advice that is available is patchy, to say the least.’*  *‘On average the UK’s wealthy population gives a score of just 5.9 out of 10 for the philanthropy advice experience they receive from their professional advisers. Yet, it is relatively straightforward – in theory at least – for professional advisers to develop the knowledge and networks to support their clients across the whole philanthropy advice process.’*  *‘… a philanthropy adviser … can guide you to learn more and meet like- minded individuals and organisations to deepen your knowledge and understanding of how your resources can make a difference. ‘*  Theme: Philanthropy Advisors  Theme: Philanthropy Consultancies | Study exploring why the UK’s HNWIs and UHNWIs give so little in the context of their overall wealth. | UK | 503 participants drawn from UK all HNWIs also sampled 383 professional services firms and interviewed 22 people working in the philanthropy sector. |

| BARRIERS TO THE USE OF EVIDENCE **Finding 6: A broader definition of what counts as credible evidence and better standardisation of reporting will facilitate the use of evidence (3/9 studies)** | | | | |
| --- | --- | --- | --- | --- |
| **STUDY** | DATA FROM THE INDIVIDUAL STUDIES THAT CONTRIBUTED TO THE FINDING | STUDY AIM | STUDY SETTING | STUDY PARTICIPANTS |
| **Study 5**  **2018** | Study refers to the fact that the standardisation of reporting would help to improve the uptake of evidence but points out that whilst ‘*New methodologies … are emerging … none is yet viewed as a panacea.’*  The study cautions that ‘*in the pursuit of hard, empirical data, individuals and their stories may be lost in a flurry of numbers. But these stories are a form of knowledge too…’* | Qualitative study which seeks to capture an overview of the collective debate on the future of philanthropy. | Dubai, Ecuador, India, Malaysia, Singapore, UK and USA, | 200 + participants drawn from academia, business, government, advisory, charities and NGOs. |
| **Study 7**  **2013** | Study raised the question as to what counts as ‘evidence’ and what counts as ‘good enough’ evidence.  Evidence and knowledge come in many guises.  *‘It is clear that funders have a wealth of experience and knowledge that they bring to bear during the funding process. Much of this is tacit and relational based on what and who they know’.* | Discussion paper exploring how a small group of UK funders use and share evidence in practice. What evidence they draw on, how they find and use it. And how they share evidence to inform the future decisions of others – funders, practitioners, policymakers. | UK | Focus of the study is on the experiences of grant-making trusts and foundations. |
| **Study 9**  **2011** | Study | This study explored the extent to which knowledge is shared across the third sector and also sought to highlight examples of good practice to share with funders. | UK | 12 participants from UK foundations. |

## **Table 7: CER-Qual – Quality of Evidence Table**

| CERQual Quality of Evidence Profile | | | | | | | |
| --- | --- | --- | --- | --- | --- | --- | --- |
| REVIEW FINDING | STUDIES CONTRIBUTING TO THE REVIEW FINDING | ASSESSMENT OF METHODOLOGICAL LIMITATIONS | ASSESSMENT OF RELEVANCE | ASSESSMENT OF COHERENCE | ASSESSMENT OF ADEQUACY | OVERALL CER-Qual ASSESSMENT OF CONFIDENCE | EXPLANATION OF JUDGMENT |
| 1. Philanthropists and third sector professionals encounter difficulties in accessing evidence to inform their decision making | 1, 2, 3, 5, 7 & 9 | Moderate methodological limitations. Only one of the included studies contained an appropriate description of the researcher reflexivity and none of them reported on whether ethics approval had been sought or granted. In 5 out of the 6 studies the method of data analysis is not described. | No or very minor concerns about relevance. | Only minor concerns about coherence. | No or very minor concerns about adequacy. | Moderate confidence | The finding was graded as moderate confidence because of some concerns regarding methodological limitations. |
| 1. Philanthropists and third sector professionals experience challenges in understanding evidence. | 1, 2, 5, 7 & 8 | Moderate methodological limitations. Only one of the included studies contained an appropriate description of the researcher reflexivity and none of them reported on whether ethics approval had been sought or granted. In 4 out of the 5 studies the method of data analysis is not described. | No or very minor concerns about relevance. | Only minor concerns about coherence. | No or very minor concerns about adequacy. | Moderate confidence | The finding was graded as moderate confidence because of some concerns regarding methodological limitations. |
| 1. Philanthropists and third sector professionals have insufficient resources to utilise evidence. | 1, 2, 5, 7, 8 & 9 | Moderate methodological limitations. Only one of the included studies contained an appropriate description of the researcher reflexivity and none of them reported on whether ethics approval had been sought or granted. In 5 out of the 6 studies the method of data analysis is not described. | No or very minor concerns about relevance. | Only minor concerns about coherence. | No or very minor concerns about adequacy. | Moderate confidence | The finding was graded as moderate confidence because of some concerns regarding methodological limitations. |
| 1. Knowledge transfer and ease of access facilitates the use of evidence. | 2, 3, 5, 6, 7 & 8 | Moderate methodological limitations. Only one of the included studies contained an appropriate description of the researcher reflexivity and none of them reported on whether ethics approval had been sought or granted. In 5 out of the 6 studies the method of data analysis is not described. | No or very minor concerns about relevance. | Only minor concerns about coherence. | No or very minor concerns about adequacy. | Moderate confidence | The finding was graded as moderate confidence because of some concerns regarding methodological limitations. |
| 1. Professional advisors facilitate the uptake of evidence | 2, 7 & 9 | Moderate methodological limitations. Only one of the included studies contained an appropriate description of the researcher reflexivity and none of them reported on whether ethics approval had been sought or granted. In none of the studies is the method of data analysis described. | No or very minor concerns about relevance. | Only minor concerns about coherence. | No or very minor concerns about adequacy. | Moderate confidence | The finding was graded as moderate confidence because of some concerns regarding methodological limitations. |
| 1. A broader definition of what counts as credible evidence and better standardisation of reporting will facilitate the use of evidence. | 2, 5, 7 & 9 | Moderate methodological limitations. Only one of the included studies contained an appropriate description of the researcher reflexivity and none of them reported on whether ethics approval had been sought or granted. In none of the studies is the method of data analysis described. | No or very minor concerns about relevance. | Only minor concerns about coherence. | No or very minor concerns about adequacy. | Moderate confidence | The finding was graded as moderate confidence because of some concerns regarding methodological limitations. |

## **Table 8: Summary of Qualitative Findings Table**

| SUMMARY OF QUALITATIVE FINDINGS TABLE | | | |
| --- | --- | --- | --- |
| Objective: To identify, evaluate and synthesize appropriate, rigorous research, examining factors which serve to act as barriers to, or facilitators of, the use of evidence by philanthropists.  Perspective: Experiences and attitudes of philanthropists, high net worth individuals and funders. | | | |
| REVIEW FINDING | CER-Qual ASSESSMENT OF CONFIDENCE IN THE EVIDENCE | EXPLANATION OF CER-Qual ASSESSMENT | STUDIES CONTRIBUTING TO THE REVIEW FINDING |
| 1. Philanthropists and third sector professionals encounter difficulties in accessing evidence to inform their decision making | Moderate confidence | The finding was graded as moderate confidence because of some concerns regarding methodological limitations. | 1, 2, 3, 5, 7 & 9 |
| 1. Philanthropists and third sector professionals experience challenges in understanding evidence. | Moderate confidence | The finding was graded as moderate confidence because of some concerns regarding methodological limitations. | 1, 2, 5, 7 & 8 |
| 1. Philanthropists and third sector professionals have insufficient resources to utilise evidence. | Moderate confidence | The finding was graded as moderate confidence because of some concerns regarding methodological limitations. | 1, 2, 5, 7, 8 & 9 |
| 1. Knowledge transfer and ease of access facilitates the use of evidence. | Moderate confidence | The finding was graded as moderate confidence because of some concerns regarding methodological limitations. | 2, 3, 5, 6, 7 & 8 |
| 1. Professional advisors facilitate the uptake of evidence | Moderate confidence | The finding was graded as moderate confidence because of some concerns regarding methodological limitations. | 2, 7 & 9 |
| 1. A broader definition of what counts as credible evidence and better standardisation of reporting will facilitate the use of evidence. | Moderate confidence | The finding was graded as moderate confidence because of some concerns regarding methodological limitations. | 2, 5, 7 & 9 |

**Table 9: Summary of Confidence in Individual Studies**

| STUDY | ASSESSMENT OF METHODOLOGICAL LIMITATIONS | ASSESSMENT OF RELEVANCE | ASSESSMENT OF COHERENCE | ASSESSMENT OF ADEQUACY | OVERALL CER-Qual ASSESSMENT OF CONFIDENCE | EXPLANATION OF JUDGMENT |
| --- | --- | --- | --- | --- | --- | --- |
| STUDY 1 | High confidence although researcher reflexivity was not described. | High confidence participants were donors to charities. | High Confidence | High Confidence | High confidence | The finding was graded as high confidence because only minor concerns relating to methodological assessment namely no description of researcher reflexivity. |
| STUDY 2 | Moderate confidence some methodological concerns – data analysis not clearly described – no researcher reflexivity described. | Moderate confidence – 40 participants drawn from academia and research | Moderate Confidence | Low Confidence | Low confidence | The finding was graded as low confidence because of some concerns regarding methodological limitations and assessment of adequacy. |
| STUDY 3 | Moderate confidence – methods of data analysis not described – no researcher reflexivity | Moderate confidence – participants worked in philanthropy and also engaged with 10 non-profit organisations. | High Confidence | High Confidence | Moderate confidence | The finding was graded as moderate confidence because of some concerns regarding methodological limitations and relevance. |
| STUDY 4 | Moderate confidence – data analysis not described in the study – no researcher reflexivity. | High confidence – Survey of 3254 US domiciled participants who give to charity and itemise charitable deductions on their tax return. | High Confidence | High Confidence | Moderate confidence | The finding was graded as high confidence because only minor concerns relating to methodological assessment namely no description of researcher reflexivity. |
| STUDY 5 | Moderate confidence – data analysis not described in detail – no researcher reflexivity. | Moderate confidence – workshops with 200+ participant ‘experts’ drawn from academia, business, government, advisory charities and NGOS. No definition of what qualifies someone as an expert. | High Confidence | Low Confidence | Moderate confidence | The finding was graded as moderate confidence because of some concerns regarding methodological limitations assessment of adequacy. |
| STUDY 6 | Moderate confidence methods of data analysis not described, no researcher reflexivity. | Low Confidence. Summarised findings from a project managed by New Philanthropy Capital examining how philanthropists are encouraged or discouraged in their giving. | Moderate Confidence | Moderate Confidence | Low confidence | The finding was graded as low confidence because of some concerns regarding methodological limitations and relevance. |
| STUDY 7 | Moderate confidence methods of data analysis not described, no researcher reflexivity. | High Confidence. Qualitative study comprising semi-structured interviews with small group of UK funders examining how they use and share evidence in practice. | High Confidence | Moderate Confidence | Moderate confidence | The finding was graded as moderate confidence because of some concerns regarding methodological limitations. |
| STUDY 8 | High Confidence. Moderate amount of author reflexivity and although the methods of data analysis were not reported in the paper the author of the study responded by email to the authors of this review to describe their methods of data collection and analysis. | High Confidence – study sampled 500 of UK’s HNWIs alongside 383 professional advisory firms offering philanthropy advice. | High Confidence | High Confidence derived from supporting documentation sent through by the author. | High Confidence | The finding was graded as high confidence because supporting documentation sent by author demonstrated methodological rigour. |
| STUDY 9 | Low Confidence. Methods of data analysis not described, no researcher reflexivity unclear if sampling method appropriate or not. | Moderate Confidence research examined extent to which knowledge is shared across the third sector. | Moderate Confidence | Moderate Confidence | Low confidence | The finding was graded as low confidence because of concerns regarding methodological limitations and relevance. |
