# Supplementary material for: A systematic review of the barriers to and facilitators of the use of evidence by philanthropists when determining which charities (including health charities or programmes) to fund
Source: Syst Rev. 2020 Aug 27;9:199. doi: 10.1186/s13643-020-01448-w (PMC7453541; doi:10.1186/s13643-020-01448-w)
Supplement: Supplementary file 4 — Additional file 4. Search Strategy. [file 13643_2020_1448_MOESM4_ESM.docx]

# File .4.

## Search Strategy Employed by: A systematic review of the barriers to and facilitators of the use of evidence utilised by philanthropists when determining which charities or programmes to fund. Caroline Greenhalgh, Paul Montgomery

Search String Employed:

[noft(barrier* OR challenge* OR constrain* OR difficult* OR interfer* OR obstruct* OR problem* OR restrain* OR restrict* OR disincentive* OR factor* OR block*) AND noft(facilitate* OR facilitator OR benefit* OR enhanc* OR influen* OR motivat* OR promot* OR aid* OR catalyst* OR Enable* OR Enhance* OR Expedite* OR Help* OR Initiat* OR Mediator*) AND noft(evidence OR knowledge) AND noft(philanthropy OR philanthropist*) AND stype.exact("Conference Papers & Proceedings" OR "Newspapers" OR "Encyclopedias & Reference Works" OR "Reports" OR "Books" OR "Working Papers" OR "Blogs, Podcasts, & Websites" OR "Scholarly Journals" OR "Dissertations & Theses") AND at.exact("Book Chapter" OR "Research Topic" OR "Dissertation/Thesis" OR "Front Page/Cover Story" OR "Government & Official Document" OR "Working Paper/Pre-Print" OR "Literature Review" OR "Biography" OR "Front Matter" OR "Conference Paper" OR "Book" OR "Conference" OR "Transcript" OR "Report" OR "Reference Document" OR "Review" OR "Case Study" OR "General Information") AND la.exact("English")](https://search.proquest.com/myresearch/savedsearches.checkdbssearchlink:rerunsearch/1551213/SavedSearches?t:ac=SavedSearches)

Saved: 30 March 2019

The following databases were searched:

1. ABI/INFORM Global
2. Applied Social Sciences Index and Abstracts (ASSIA)
3. International Bibliography of the Social Sciences (IBSS)
4. PAIS Index
5. Policy File Index
6. Social Services Abstracts
7. Social Science Premium Collection
8. Worldwide Political Science Abstracts
9. SCOPUS
10. Open Grey
11. ProQuest Dissertations & Theses Global
12. **Search Strategy for ABI/INFORM 04.06.2019**

**Returned 47 studies of which one read in full (46 excluded after reading title and abstract)**

| **Set#** | **Searched for** | **Databases** | **Results** |  | |
| --- | --- | --- | --- | --- | --- |
| S1 | [noft(barrier* OR challenge* OR constrain* OR difficult* OR interfer* OR obstruct* OR problem* OR restrain* OR restrict* OR disincentive* OR factor* OR block*) AND noft(facilitate* OR facilitator OR benefit* OR enhanc* OR influen* OR motivat* OR promot* OR aid* OR catalyst* OR Enable* OR Enhance* OR Expedite* OR Help* OR Initiat* OR Mediator*) AND noft(evidence*) AND noft(philanthropy OR philanthropist*) AND stype.exact("Conference Papers & Proceedings" OR "Working Papers" OR "Scholarly Journals" OR "Dissertations & Theses") AND at.exact("Book Chapter" OR "Annual Report" OR "Dissertation/Thesis" OR "Government & Official Document" OR "Literature Review" OR "Conference Paper" OR "Book" OR "Conference" OR "Report" OR "Statistics/Data Report" OR "Reference Document" OR "Case Study" OR "Conference Proceeding" OR "Article") AND la.exact("English")](https://search-proquest-com.ezproxye.bham.ac.uk/myresearch/savedsearches.checkdbssearchlink:rerunsearch/1584826/SavedSearches?site=pais&t:ac=SavedSearches) | ABI/INFORM Global, Worldwide Political Science Abstracts | 47 | |  |

1. **Search Strategy for Applied Social Sciences Index and Abstracts (ASSIA) 15.06.2019**

**Returned 9 studies**

| **Set#** | **Searched for** | **Databases** | **Results** |  | |
| --- | --- | --- | --- | --- | --- |
| S2 | [noft((barrier* OR challenge* OR constrain* OR difficult* OR interfer* OR obstruct* OR problem* OR restrain* OR restrict* OR disincentive* OR factor* OR block*)) AND noft((facilitate* OR facilitator OR benefit* OR enhanc* OR influen* OR motivat* OR promot* OR aid* OR catalyst* OR Enable* OR Enhance* OR Expedite* OR Help* OR Initiat* OR Mediator*)) AND noft((evidence OR knowledge)) AND noft((philanthropy OR philanthropist*)) AND stype.exact("Magazines" OR "Scholarly Journals" OR "Dissertations & Theses") AND at.exact("Report" OR "Dissertation/Thesis" OR "Statistics/Data Report" OR "Case Study" OR "Conference Paper" OR "Book" OR "Article") AND la.exact("English")](https://search-proquest-com.ezproxye.bham.ac.uk/myresearch/savedsearches.checkdbssearchlink:rerunsearch/1693117/SavedSearches?site=pais&t:ac=SavedSearches) | Applied Social Sciences Index and Abstracts (ASSIA) | 9 | |  |

1. **Search Strategy for International Bibliography of the Social Sciences (IBSS) 04.06.2019**

**Returned 36 articles of which 6 duplicates leaving 30**

| S3 | [noft(barrier* OR challenge* OR constrain* OR difficult* OR interfer* OR obstruct* OR problem* OR restrain* OR restrict* OR disincentive* OR factor* OR block*) AND noft(facilitate* OR facilitator OR benefit* OR enhanc* OR influen* OR motivat* OR promot* OR aid* OR catalyst* OR Enable* OR Enhance* OR Expedite* OR Help* OR Initiat* OR Mediator*) AND noft(evidence) AND noft(philanthropy OR philanthropist*) AND stype.exact("Newspapers" OR "Reports" OR "Books" OR "Scholarly Journals") AND at.exact("Book Chapter" OR "Front Page/Cover Story" OR "Government & Official Document" OR "Working Paper/Pre-Print" OR "Literature Review" OR "Biography" OR "Front Matter" OR "Conference Paper" OR "Book" OR "Conference" OR "Feature" OR "Transcript" OR "Report" OR "Review" OR "Case Study" OR "General Information") AND la.exact("English")](https://search-proquest-com.ezproxye.bham.ac.uk/myresearch/savedsearches.checkdbssearchlink:rerunsearch/1584807/SavedSearches?site=pais&t:ac=SavedSearches) | International Bibliography of the Social Sciences (IBSS) | 30 |
| --- | --- | --- | --- |

1. **Search Strategy for PAIS Index 15.06.2019**

**Returned 7 studies**

| **Set#** | **Searched for** | **Databases** | **Results** |  | |
| --- | --- | --- | --- | --- | --- |
| S4 | [noft((barrier* OR challenge* OR constrain* OR difficult* OR interfer* OR obstruct* OR problem* OR restrain* OR restrict* OR disincentive* OR factor* OR block*)) AND noft((facilitate* OR facilitator OR benefit* OR enhanc* OR influen* OR motivat* OR promot* OR aid* OR catalyst* OR Enable* OR Enhance* OR Expedite* OR Help* OR Initiat* OR Mediator*)) AND noft((evidence OR knowledge)) AND noft((philanthropy OR philanthropist*)) AND la.exact("English") AND at.exact("Book Chapter" OR "Report" OR "Bibliography" OR "Statistics/Data Report" OR "Working Paper/Pre-Print" OR "Conference Paper" OR "Book") AND stype.exact("Conference Papers & Proceedings" OR "Reports" OR "Books" OR "Scholarly Journals")](https://search-proquest-com.ezproxye.bham.ac.uk/myresearch/savedsearches.checkdbssearchlink:rerunsearch/1693118/SavedSearches?site=pais&t:ac=SavedSearches) | PAIS Index | 7 | |  |

1. **Search Strategy for Policy File Index 15.06.2019**

**Returned 7 studies including 1 duplicate**

| **Set#** | **Searched for** | **Databases** | **Results** |  | |
| --- | --- | --- | --- | --- | --- |
| S5 | Searched for:  [noft((barrier* OR challenge* OR constrain* OR difficult* OR interfer* OR obstruct* OR problem* OR restrain* OR restrict* OR disincentive* OR factor* OR block*)) AND noft((facilitate* OR facilitator OR benefit* OR enhanc* OR influen* OR motivat* OR promot* OR aid* OR catalyst* OR Enable* OR Enhance* OR Expedite* OR Help* OR Initiat* OR Mediator*)) AND noft((evidence OR knowledge)) AND noft((philanthropy OR philanthropist*)) AND rtype.exact("Report") AND la.exact("English")](https://search-proquest-com.ezproxye.bham.ac.uk/myresearch/savedsearches.checkdbssearchlink:rerunsearch/1693120/SavedSearches?site=pais&t:ac=SavedSearches) | Policy File Index | 7 | |  |

1. **Search Strategy for Social Services Abstracts 15.06.2019**

**Returned 1 study**

| **Set#** | **Searched for** | **Databases** | **Results** |  | |
| --- | --- | --- | --- | --- | --- |
| S6 | Searched for:  [noft((barrier* OR challenge* OR constrain* OR difficult* OR interfer* OR obstruct* OR problem* OR restrain* OR restrict* OR disincentive* OR factor* OR block*)) AND noft((facilitate* OR facilitator OR benefit* OR enhanc* OR influen* OR motivat* OR promot* OR aid* OR catalyst* OR Enable* OR Enhance* OR Expedite* OR Help* OR Initiat* OR Mediator*)) AND noft((evidence OR knowledge)) AND noft((philanthropy OR philanthropist*)) AND at.exact("Report" OR "Dissertation/Thesis" OR "Statistics/Data Report" OR "Working Paper/Pre-Print" OR "Literature Review" OR "Review" OR "Case Study" OR "Editorial" OR "Conference Paper" OR "Book") AND la.exact("English")](https://search-proquest-com.ezproxye.bham.ac.uk/myresearch/savedsearches.checkdbssearchlink:rerunsearch/1693122/SavedSearches?site=pais&t:ac=SavedSearches) | Social Services Abstracts | 1 | |  |

1. **Search Strategy for Social Science Premium Collection 15.06.2019**

**Returned 287 studies – after title screening 15 screened by their abstracts**

| **Set#** | **Searched for** | **Databases** | **Results** |  | |
| --- | --- | --- | --- | --- | --- |
| S7 | noft(barrier* OR challenge* OR constrain* OR difficult* OR interfer* OR obstruct* OR problem* OR restrain* OR restrict* OR disincentive* OR factor* OR block*) AND noft(facilitate* OR facilitator OR benefit* OR enhanc* OR influen* OR motivat* OR promot* OR aid* OR catalyst* OR Enable* OR Enhance* OR Expedite* OR Help* OR Initiat* OR Mediator*) AND noft(evidence OR knowledge) AND noft(philanthropy OR philanthropist*) AND at.exact("Book Chapter" OR "Research Topic" OR "Dissertation/Thesis" OR "Front Page/Cover Story" OR "Government & Official Document" OR "Working Paper/Pre-Print" OR "Literature Review" OR "Biography" OR "Front Matter" OR "Conference Paper" OR "Book" OR "Conference" OR "Transcript" OR "Report" OR "Reference Document" OR "Review" OR "Case Study" OR "General Information") AND la.exact("English") AND stype.exact("Books" OR "Working Papers" OR "Scholarly Journals" OR "Dissertations & Theses") | Social Science Premium Collection | 287 | |  |

1. **Search Strategy for Worldwide Political Science Abstracts 15.06.2019**

**Returned 6 studies**

| **Set#** | **Searched for** | **Databases** | **Results** |  | |
| --- | --- | --- | --- | --- | --- |
| S8 | [noft(barrier* OR challenge* OR constrain* OR difficult* OR interfer* OR obstruct* OR problem* OR restrain* OR restrict* OR disincentive* OR factor* OR block*) AND noft(facilitate* OR facilitator OR benefit* OR enhanc* OR influen* OR motivat* OR promot* OR aid* OR catalyst* OR Enable* OR Enhance* OR Expedite* OR Help* OR Initiat* OR Mediator*) AND noft(evidence OR knowledge) AND noft(philanthropy OR philanthropist*) AND at.exact("Book Chapter" OR "Research Topic" OR "Dissertation/Thesis" OR "Front Page/Cover Story" OR "Government & Official Document" OR "Working Paper/Pre-Print" OR "Literature Review" OR "Biography" OR "Front Matter" OR "Conference Paper" OR "Book" OR "Conference" OR "Transcript" OR "Report" OR "Reference Document" OR "Review" OR "Case Study" OR "General Information") AND la.exact("English") AND stype.exact("Books" OR "Working Papers" OR "Scholarly Journals" OR "Dissertations & Theses")](https://search-proquest-com.ezproxye.bham.ac.uk/myresearch/savedsearches.checkdbssearchlink:rerunsearch/1590434/SavedSearches?site=pais&t:ac=SavedSearches) | Worldwide Political Science Abstracts | 6 | |  |

1. **Search Strategy for SCOPUS 15.06.2019**

**Returned 124 studies – after reading titles 6 were screened at abstract**

| **Set#** | **Searched for** | **Databases** | **Results** |  | |
| --- | --- | --- | --- | --- | --- |
| S9 | TITLE-ABS-KEY ( barrier*  OR  challenge*  OR  constrain*  OR  difficult*  OR  interfer*  OR  obstruct*  OR  problem*  OR  restrain*  OR  restrict*  OR  disincentive*  OR  factor*  OR  block* )  AND  TITLE-ABS-KEY ( facilitate*  OR  facilitator  OR  benefit*  OR  enhanc*  OR  influen*  OR  motivat*  OR  promot*  OR  aid*  OR  catalyst*  OR  enable*  OR  enhance*  OR  expedite*  OR  help*  OR  initiat*  OR  mediator* )  AND  TITLE-ABS-KEY ( evidence  OR  knowledge )  AND  TITLE-ABS-KEY ( philanthropy  OR  philanthropist* ) ) | SCOPUS | 124 | |  |

1. **Search Strategy for Open Grey 15.06.2019**

**Returned studies**

| **Set#** | **Searched for** | **Databases** | **Results** |  | |
| --- | --- | --- | --- | --- | --- |
| S8 | [noft(barrier* OR challenge* OR constrain* OR difficult* OR interfer* OR obstruct* OR problem* OR restrain* OR restrict* OR disincentive* OR factor* OR block*) AND noft(facilitate* OR facilitator OR benefit* OR enhanc* OR influen* OR motivat* OR promot* OR aid* OR catalyst* OR Enable* OR Enhance* OR Expedite* OR Help* OR Initiat* OR Mediator*) AND noft(evidence OR knowledge) AND noft(philanthropy OR philanthropist*) AND at.exact("Book Chapter" OR "Research Topic" OR "Dissertation/Thesis" OR "Front Page/Cover Story" OR "Government & Official Document" OR "Working Paper/Pre-Print" OR "Literature Review" OR "Biography" OR "Front Matter" OR "Conference Paper" OR "Book" OR "Conference" OR "Transcript" OR "Report" OR "Reference Document" OR "Review" OR "Case Study" OR "General Information") AND la.exact("English") AND stype.exact("Books" OR "Working Papers" OR "Scholarly Journals" OR "Dissertations & Theses")](https://search-proquest-com.ezproxye.bham.ac.uk/myresearch/savedsearches.checkdbssearchlink:rerunsearch/1590434/SavedSearches?site=pais&t:ac=SavedSearches) | Worldwide Political Science Abstracts | 6 | |  |

**Search Strategy for Open Grey 15.06.2019**

**Returned 0 studies**

| **Set#** | **Searched for** | **Databases** | **Results** |  | |
| --- | --- | --- | --- | --- | --- |
| S10 | (barrier* OR challenge* OR constrain* OR difficult* OR interfer* OR obstruct* OR problem* OR restrain* OR restrict* OR disincentive* OR factor* OR block*) AND (facilitate* OR facilitator OR benefit* OR enhanc* OR influen* OR motivat* OR promot* OR aid* OR catalyst* OR Enable* OR Enhance* OR Expedite* OR Help* OR Initiat* OR Mediator*) AND (evidence OR knowledge) AND noft(philanthropy OR philanthropist*) | Open Grey | 0 | |  |

**Search Strategy for ProQuest Dissertations & Theses Global**

**Returned 111 studies**

| **Set#** | **Searched for** | **Databases** | **Results** |  | |
| --- | --- | --- | --- | --- | --- |
| S11 | [noft((barrier* OR challenge* OR constrain* OR difficult* OR interfer* OR obstruct* OR problem* OR restrain* OR restrict* OR disincentive* OR factor* OR block*)) AND noft((facilitate* OR facilitator OR benefit* OR enhanc* OR influen* OR motivat* OR promot* OR aid* OR catalyst* OR Enable* OR Enhance* OR Expedite* OR Help* OR Initiat* OR Mediator*)) AND noft((evidence OR knowledge)) AND noft((philanthropy OR philanthropist*)) AND stype.exact("Magazines" OR "Scholarly Journals" OR "Dissertations & Theses") AND at.exact("Report" OR "Dissertation/Thesis" OR "Statistics/Data Report" OR "Case Study" OR "Conference Paper" OR "Book" OR "Article") AND la.exact("English")](https://search-proquest-com.ezproxye.bham.ac.uk/myresearch/savedsearches.checkdbssearchlink:rerunsearch/1693117/SavedSearches?site=pais&t:ac=SavedSearches) | ProQuest Dissertations & Theses Global | 111 | |  |

111 returned studies – 2 duplicates = 109 studies after screening titles 2 abstracts were read.

## **List of 32 studies drawn from database search read in full after title and abstract Screening**

1. [Reconciling Community-Based Versus Evidence-Based Philanthropy: A Case Study of The Colorado Trust's Early Initiatives](https://search-proquest-com.ezproxyd.bham.ac.uk/socialsciencepremium/docview/1838687674/8AEB25D5B70B496APQ/1?accountid=8630)

Easterling, Douglas, PhD; Main, Deborah, PhD.**The Foundation Review; Grand Rapids** Vol. 8, Iss. 4 : 81-107,110.

1. [Nonprofits and evaluation: Empirical evidence from the field](https://search-proquest-com.ezproxyd.bham.ac.uk/socialsciencepremium/docview/61774430/8AEB25D5B70B496APQ/2?accountid=8630)

Carman, Joanne G; Fredericks, Kimberly A. **New Directions for Evaluation** Iss. 119 (October 2008): 51-71.

1. [Evaluating Nonprofit Databases](https://search-proquest-com.ezproxyd.bham.ac.uk/socialsciencepremium/docview/60428051/8AEB25D5B70B496APQ/3?accountid=8630)

Gronbjerg, Kirsten A. **American Behavioral Scientist** Vol. 45, Iss. 11,  (July 2002): 1741-1777.

1. [How Social Entrepreneurs in the Third Sector Learn from Life Experiences](https://search-proquest-com.ezproxyd.bham.ac.uk/socialsciencepremium/docview/1811140764/8AEB25D5B70B496APQ/4?accountid=8630)

Scheiber, Laura.**Voluntas; Baltimore** Vol. 27, Iss. 4,  (Aug 2016): 1694-1717.

1. [Addressing Deep and Persistent Poverty: A Framework for Philanthropic Planning and Investment](https://search-proquest-com.ezproxyd.bham.ac.uk/socialsciencepremium/docview/1537585956/8AEB25D5B70B496APQ/5?accountid=8630)

Aron, Laudan; Jacobson, Wendy; Turner, Margery Austin.*Urban Institute, Dec 2013, 32 pp*. Urban Institute, 2013.

1. [Philanthropic Motivation in the 21st Century](https://search-proquest-com.ezproxyd.bham.ac.uk/socialsciencepremium/docview/1648677263/8AEB25D5B70B496APQ/6?accountid=8630)

Dolan, John F.Drexel University, Drexel University. 3669449.

1. [Why philanthropy matters: how the wealthy give, and what it means for our economic well-being](https://search-proquest-com.ezproxyd.bham.ac.uk/socialsciencepremium/docview/1418111937/8AEB25D5B70B496APQ/7?accountid=8630)

Acs, Zoltan J.xv, 249. Princeton NJ: Princeton University Press, 2013.

1. [Gifts on a High Note: A Case Study of Major Donors to Music Programs in Higher Education](https://search-proquest-com.ezproxyd.bham.ac.uk/socialsciencepremium/docview/1373090582/8AEB25D5B70B496APQ/8?accountid=8630)

Barascout, Roger.

1. [The role of evaluations in community foundations](https://search-proquest-com.ezproxyd.bham.ac.uk/socialsciencepremium/docview/1322726205/8AEB25D5B70B496APQ/9?accountid=8630)

Watts, Brad R.AAI3492984.

1. [The New Altruism: Patterns of Private Giving and the Transformation of Welfare Solidarity](https://search-proquest-com.ezproxyd.bham.ac.uk/socialsciencepremium/docview/61781054/8AEB25D5B70B496APQ/10?accountid=8630)

Bode, Ingo; Brose, Hanns-Georg.**International Sociological Association**  (0, 1998)

1. [Searching for Enduring Donor Relationships: Evidence for Factors and Strategies in a Donor/Organization Integration Model for Fund Raising](https://search-proquest-com.ezproxyd.bham.ac.uk/socialsciencepremium/docview/881467072/8AEB25D5B70B496APQ/11?accountid=8630)

Chung-Hoon, Tanise L; Hite, Julie M; Hite, Steven J.**International Journal of Educational Advancement** Vol. 6, Iss. 1,  (Nov 2005): 34-53.

1. [Data for Good: Unlocking Privately-Held Data to the Benefit of the Many](https://search-proquest-com.ezproxyd.bham.ac.uk/socialsciencepremium/docview/2072931104/8AEB25D5B70B496APQ/12?accountid=8630)

Alemanno, Alberto.**IDEAS Working Paper Series from RePEc; St. Louis**, 2018.

1. [An Examination of Motivational Factors Affecting African American Alumni Philanthropy at a Historically Black University](https://search-proquest-com.ezproxyd.bham.ac.uk/socialsciencepremium/docview/1916583453/8AEB25D5B70B496APQ/13?accountid=8630)

Beamon, Ann McCabe.North Carolina Agricultural and Technical State University, North Carolina Agricultural and Technical State University. 10264412.

1. [The Rising Price of Objectivity: Philanthropy, Government, and the Future of Education Research](https://search-proquest-com.ezproxyd.bham.ac.uk/socialsciencepremium/docview/1871572294/8AEB25D5B70B496APQ/14?accountid=8630)

Feuer, Michael J; Harvard University, Graduate School of Education.*Harvard Education Press*. 208. Harvard Education Press, 8 Story Street First Floor, Cambridge, MA 02138, 2016.

1. [Moving the Needle: What Works Cities and the use of data and evidence](https://search-proquest-com.ezproxyd.bham.ac.uk/socialsciencepremium/docview/2080371404/8AEB25D5B70B496APQ/15?accountid=8630)

Brody, Simone; Koester, Andel; Markovits, Zachary; Phillips, Jacob.**arXiv.org; Ithaca**, 2016.

1. [A Case Study on Moving Philosophically Diverse Funders to Common Priorities. Education Funders Research Initiative](https://search-proquest-com.ezproxyd.bham.ac.uk/socialsciencepremium/docview/1871568262/8AEB25D5B70B496APQ/16?accountid=8630)

Hilliard, Thomas J; Education Funders Research Initiative; Center for an Urban Future; Philanthropy New York.**Education Funders Research Initiative**, (Jul 2015).

1. [Giving to Excellence: Generating Philanthropic Support for UK Higher Education. Ross-CASE Report 2016](https://search-proquest-com.ezproxyd.bham.ac.uk/socialsciencepremium/docview/1968427391/8AEB25D5B70B496APQ/17?accountid=8630)

Jain, Yashraj; Council for Advancement and Support of Education.**Council for Advancement and Support of Education**, (Apr 26, 2016).

1. [Women and Philanthropy in Higher Education: A Collective Case Study of Major Donors](https://search-proquest-com.ezproxyd.bham.ac.uk/socialsciencepremium/docview/1803233769/8AEB25D5B70B496APQ/18?accountid=8630)

Lahti Tunnell, Michele Lynn.University of the Pacific, University of the Pacific. 10117036.

1. [Philanthropy & policy change: Exploring the role of private charitable foundations in the policymaking process](https://search-proquest-com.ezproxyd.bham.ac.uk/socialsciencepremium/docview/1718065624/8AEB25D5B70B496APQ/19?accountid=8630)

Franklin, Jason.AAI3642618.

1. ["Moneyball" for Education Using Data, Evidence, and Evaluation to Improve Federal Education Policy](https://search-proquest-com.ezproxyd.bham.ac.uk/socialsciencepremium/docview/1720059874/8AEB25D5B70B496APQ/20?accountid=8630)

Hess, Frederick M; Little, Bethany; Results for America; American Enterprise Institute for Public Policy Research (AEI).**American Enterprise Institute for Public Policy Research**, (Mar 2015).

1. [Can We Identify a Successful Teacher Better, Faster, and Cheaper? Evidence for Innovating Teacher Observation Systems](https://search-proquest-com.ezproxyd.bham.ac.uk/socialsciencepremium/docview/1651852852/8AEB25D5B70B496APQ/21?accountid=8630)

Gargani, John; Strong, Michael.**Journal of Teacher Education** Vol. 65, Iss. 5,  (Nov 2014 - Dec 2014): 389-401.

1. [Funding for Change: Factors Affecting Foundation Funding of Pre-Collegiate Education Policy in the United States Following the Charlottesville Summit and No Child Left Behind](https://search-proquest-com.ezproxyd.bham.ac.uk/socialsciencepremium/docview/1682038667/8AEB25D5B70B496APQ/22?accountid=8630)

Klopott, Shayna Melinda.Columbia University, Columbia University. 3702315.

1. [Leveraging core competencies in corporate philanthropy: Cisco's exemplary strategic philanthropy](https://search-proquest-com.ezproxyd.bham.ac.uk/socialsciencepremium/docview/1524022650/8AEB25D5B70B496APQ/23?accountid=8630)

Shannon, Nancy Colleen.Capella University, Capella University. 3615448.

1. [Non-traditional aid and gender equity: Evidence from million dollar donations](https://search-proquest-com.ezproxyd.bham.ac.uk/socialsciencepremium/docview/1497407436/8AEB25D5B70B496APQ/24?accountid=8630)

Okonkwo Osili, Una.*World Institute for Development Economics Research, United Nations University, Aug 2013, 33 pp*. World Institute for Development Economics Research, United Nations University, 2013.

1. [Awaking the Public Sector with Strategic Corporate Philanthropy: Revitalizing the Public Servant's Organizational Knowledge, Innovative Capability, and Commitment](https://search-proquest-com.ezproxyd.bham.ac.uk/socialsciencepremium/docview/1322253405/8AEB25D5B70B496APQ/25?accountid=8630)

Jackson, Janese Marie.

1. [Women healthcare philanthropists: Sharing their resources with community healthcare systems](https://search-proquest-com.ezproxyd.bham.ac.uk/socialsciencepremium/docview/889930473/8AEB25D5B70B496APQ/26?accountid=8630)

Abraham, Diane M.Marian University, Marian University. 3468984.

1. [Evidence-Based Programs in Action: Policy and Practice Insights from a Success Story. Research-to-Results Brief. Publication #2010-08](https://search-proquest-com.ezproxyd.bham.ac.uk/socialsciencepremium/docview/742847035/8AEB25D5B70B496APQ/27?accountid=8630)

Uninsky, Philip; Child Trends.**Child Trends**, (Apr 2010).

1. [What Program Providers Want Researchers to Know. Research-to-Results Practitioner Insights. Publication # 2009-03](https://search-proquest-com.ezproxyd.bham.ac.uk/socialsciencepremium/docview/742863887/8AEB25D5B70B496APQ/28?accountid=8630)

Moore, Kristin Anderson; Child Trends.**Child Trends**, (Jan 2009).

1. [Benchmarking 2009: Trends in Education Philanthropy](https://search-proquest-com.ezproxyd.bham.ac.uk/socialsciencepremium/docview/1312421934/8AEB25D5B70B496APQ/29?accountid=8630)

Bearman, Jessica; Kilgore, Gin; GRANTMAKERS FOR EDUCATION.**Grantmakers for Education**,

1. [Implementing Evidence Based Practices: Six "Drivers" of Success. Part 3 in a Series on Fostering the Adoption of Evidence-Based Practices in Out-Of-School Time Programs. Research-to-Results Brief. Publication #2007-29](https://search-proquest-com.ezproxyd.bham.ac.uk/socialsciencepremium/docview/61914619/8AEB25D5B70B496APQ/30?accountid=8630)

Metz, Allison J. R; Blase, Karen; Bowie, Lillian; Child Trends, Inc., Washington, DC.**Child Trends**, (Oct 2007).

1. [CASA: Case study of a community college-based outreach program](https://search-proquest-com.ezproxyd.bham.ac.uk/socialsciencepremium/docview/305347678/8AEB25D5B70B496APQ/31?accountid=8630)

Rodriguez, Nelly.Pepperdine University, Pepperdine University. 3202403.

1. [Expert inquiry and health care reform in New Era America: Herbert Hoover, Ray Lyman Wilbur, and the travails of the disinterested experts](https://search-proquest-com.ezproxyd.bham.ac.uk/socialsciencepremium/docview/304136248/8AEB25D5B70B496APQ/32?accountid=8630)

Parks, Douglas R.The University of Iowa, The University of Iowa. 9525181.
