# Supplementary material for: A systematic review of the barriers to and facilitators of the use of evidence by philanthropists when determining which charities (including health charities or programmes) to fund
Source: Syst Rev. 2020 Aug 27;9:199. doi: 10.1186/s13643-020-01448-w (PMC7453541; doi:10.1186/s13643-020-01448-w)
Supplement: Supplementary file 5 — Additional file 5. JBI Checklists. [file 13643_2020_1448_MOESM5_ESM.pdf]

DocuSign Envelope ID: E1C22B0D-C018-41E0-8C0E-AB0EEF4C7B3A

# JBI Critical Appraisal Checklist for Qualitative Research

Reviewer Caroline Greenhalgh Date 30.06.2019

Author Beth Breeze Year 2013 Record Number ______

| | Yes | No | Unclear | Not applicable |
|---|---|---|---|---|
| 1. Is there congruity between the stated philosophical perspective and the research methodology? | ☐ | ☐ | ☒ | ☐ |
| 2. Is there congruity between the research methodology and the research question or objectives? | ☒ | ☐ | ☐ | ☐ |
| 3. Is there congruity between the research methodology and the methods used to collect data? | ☒ | ☐ | ☐ | ☐ |
| 4. Is there congruity between the research methodology and the representation and analysis of data? | ☒ | ☐ | ☐ | ☐ |
| 5. Is there congruity between the research methodology and the interpretation of results? | ☒ | ☐ | ☐ | ☐ |
| 6. Is there a statement locating the researcher culturally or theoretically? | ☐ | ☒ | ☐ | ☐ |
| 7. Is the influence of the researcher on the research, and vice- versa, addressed? | ☐ | ☒ | ☐ | ☐ |
| 8. Are participants, and their voices, adequately represented? | ☒ | ☐ | ☐ | ☐ |
| 9. Is the research ethical according to current criteria or, for recent studies, and is there evidence of ethical approval by an appropriate body? | ☐ | ☐ | ☒ | ☐ |
| 10. Do the conclusions drawn in the research report flow from the analysis, or interpretation, of the data? | ☒ | ☐ | ☐ | ☐ |

Overall appraisal: Include ☒ Exclude ☐ Seek further info ☐

Comments (Including reason for exclusion)

______________________________

______________________________

______________________________
